# Supplementary material for: Risk prediction models for hepatic encephalopathy post-transjugular intrahepatic portosystemic shunt: A systematic review and critical appraisal
Source: JHEP Rep. 2026 Aug 22;8(11):102006. doi: 10.1016/j.jhepr.2026.102006 (PMC13694966; doi:10.1016/j.jhepr.2026.102006)

Risk prediction models for hepatic encephalopathy post-transjugular intrahepatic portosystemic shunt: A systematic review and critical appraisal

Stijn de Reus, Wenjing Wang, Wichor M. Bramer, Juan-Carlos Garcia-Pagan, Anna Baiges, Bettina E Hansen, Raoel Maan

Table of contents

Table S1……………………………………………………………………………………2

Fig. S1……………………………………………………………………………………...5

Fig. S2……………………………………………………………………………………...6

Table S1. Search terms used for systematic review

**Database searched**	**Platform**	**Years of coverage**	**Records**	**Records after duplicates removed**
Medline ALL	Ovid	1946 - Present	1039	1031
Embase	Embase.com	1971 - Present	1462	645
Web of Science Core Collection*	Web of Knowledge	1975 - Present	1210	345
CINAHL Plus	EBSCO	1982 - Present	334	123
**Total**			**4045**	**2144**

*Science Citation Index Expanded (1975-present) ; Social Sciences Citation Index (1975-present) ; Arts & Humanities Citation Index (1975-present) ; Conference Proceedings Citation Index- Science (1990-present) ; Conference Proceedings Citation Index- Social Science & Humanities (1990-present) ; Emerging Sources Citation Index (2005-present)

No other database limits were used than those specified in the search strategies

Search terms

**Medline**
(Portasystemic Shunt, Transjugular Intrahepatic / OR (((transjug* OR trans-jug*) ADJ3 (intrahepat* OR intra-hepat* OR portosyst* OR port*-syst*) ADJ6 shunt*) OR post-tips OR posttips OR ptips OR post-tipss OR posttipss OR ptipss).ab,ti,kf. OR (tips OR tipss).ti.) AND (Brain Diseases / OR Hepatic Encephalopathy/ OR exp Survival / OR exp Mortality / OR (encephalopath* OR hepatocereb* OR hepatoencephalopath* OR surviv* OR mortalit*).ab,ti,kf. OR (he OR ohe).ti.) AND (Prediction Algorithms/ OR Prediction Methods, Machine/ OR Prognosis/ OR Risk/ OR Risk Factors/ OR Validation Study/ OR Nomograms/ OR exp Machine Learning/ OR Machine Learning Algorithms/ OR exp Artificial

Intelligence/ OR (predict* OR prognos* OR Risk-score* OR Risk-factor* OR risk-model* OR validation* OR nomogra* OR ((machine OR deep) ADJ learning) OR (artificial* ADJ3 intelligen*)).ab,ti,kf. OR (risk OR index OR model*).ti.) NOT (news OR congres* OR abstract* OR book* OR chapter* OR dissertation abstract*).pt. NOT (exp animals/ NOT humans/) AND english.la.
**Embase**
('transjugular intrahepatic portosystemic shunt'/de OR 'TIPS endoprosthesis'/de OR (((transjug* OR trans-jug*) NEAR/3 (intrahepat* OR intra-hepat* OR portosyst* OR port*-syst*) NEAR/6 shunt*) OR post-tips OR posttips OR ptips OR post-tipss OR posttipss OR ptipss):ab,ti,kw OR (tips OR tipss):ti) AND (encephalopathy/exp OR survival/exp OR mortality/exp OR 'mortality risk'/de OR (encephalopath* OR hepatocereb* OR hepatoencephalopath* OR surviv* OR mortalit*):ab,ti,kw OR (he OR ohe):ti) AND (prediction/exp OR prognosis/de OR risk/de OR 'risk factor'/de OR 'risk score model'/de OR 'risk score'/de OR 'risk scoring system'/de OR 'risk model'/de OR 'mortality risk'/de OR 'validation process'/de OR 'validation study'/de OR nomogram/de OR 'machine learning'/exp OR 'artificial intelligence'/exp OR (predict* OR prognos* OR Risk-score* OR Risk-factor* OR risk-model* OR validation* OR nomogra* OR ((machine OR deep) NEXT/1 learning) OR (artificial* NEAR/3 intelligen*)):ab,ti,kw OR (risk OR index OR model*):ti) NOT ([Conference Abstract]/lim OR [Conference Review]/lim OR [preprint]/lim OR 'clinical trial':it) NOT ([letter]/lim OR [editorial]/lim) NOT ([animals]/lim NOT [humans]/lim) AND [english]/lim
**Web of science**
(TS=(((transjug* OR trans-jug*) NEAR/2 (intrahepat* OR intra-hepat* OR portosyst* OR port*-syst*) NEAR/5 shunt*) OR post-tips OR posttips OR ptips OR post-tipss OR posttipss OR ptipss) OR TI=(tips OR tipss)) AND (TS=(encephalopath* OR hepatocereb* OR hepatoencephalopath* OR surviv* OR mortalit*) OR TI=(he OR ohe)) AND (TS=(predict* OR prognos* OR Risk-score* OR Risk-factor* OR risk-model* OR validation* OR nomogra* OR ((machine OR deep) NEAR/1 learning) OR (artificial* NEAR/2 intelligen*)) OR TI=(risk OR index OR model*)) NOT DT=(Meeting Abstract OR Meeting Summary) AND LA=(English)

**CINAHL**
(XB(((transjug* OR trans-jug*) N2 (intrahepat* OR intra-hepat* OR portosyst* OR port*-syst*) N5 shunt*) OR post-tips OR posttips OR ptips OR post-tipss OR posttipss OR ptipss) OR TI(tips OR tipss)) AND (MH Brain Diseases OR MH Hepatic Encephalopathy+ OR MH Survival + OR MH Mortality + OR XB(encephalopath* OR hepatocereb* OR hepatoencephalopath* OR surviv* OR mortalit*) OR TI(he OR ohe)) AND (MH Prediction Algorithms+ OR MH Prediction Algorithms + OR MH Prediction Models OR MH Predictive Validity OR MH Prognosis+ OR MH Risk+ OR MH Risk Factors+ OR MH Validation Studies + OR MH Machine Learning+ OR MH Machine Learning Algorithms+ OR MH Artificial Intelligence+ OR XB(predict* OR prognos* OR Risk-score* OR Risk-factor* OR risk-model* OR validation* OR nomogra* OR ((machine OR deep) N1 learning) OR (artificial* N2 intelligen*)) OR TI(risk OR index OR model*)) NOT PT(news OR congres* OR abstract* OR book* OR chapter* OR dissertation abstract*) NOT (MH animals+ NOT MH humans) AND LA(english)

Fig. S1 PRISMA 2020 flow diagram of study selection

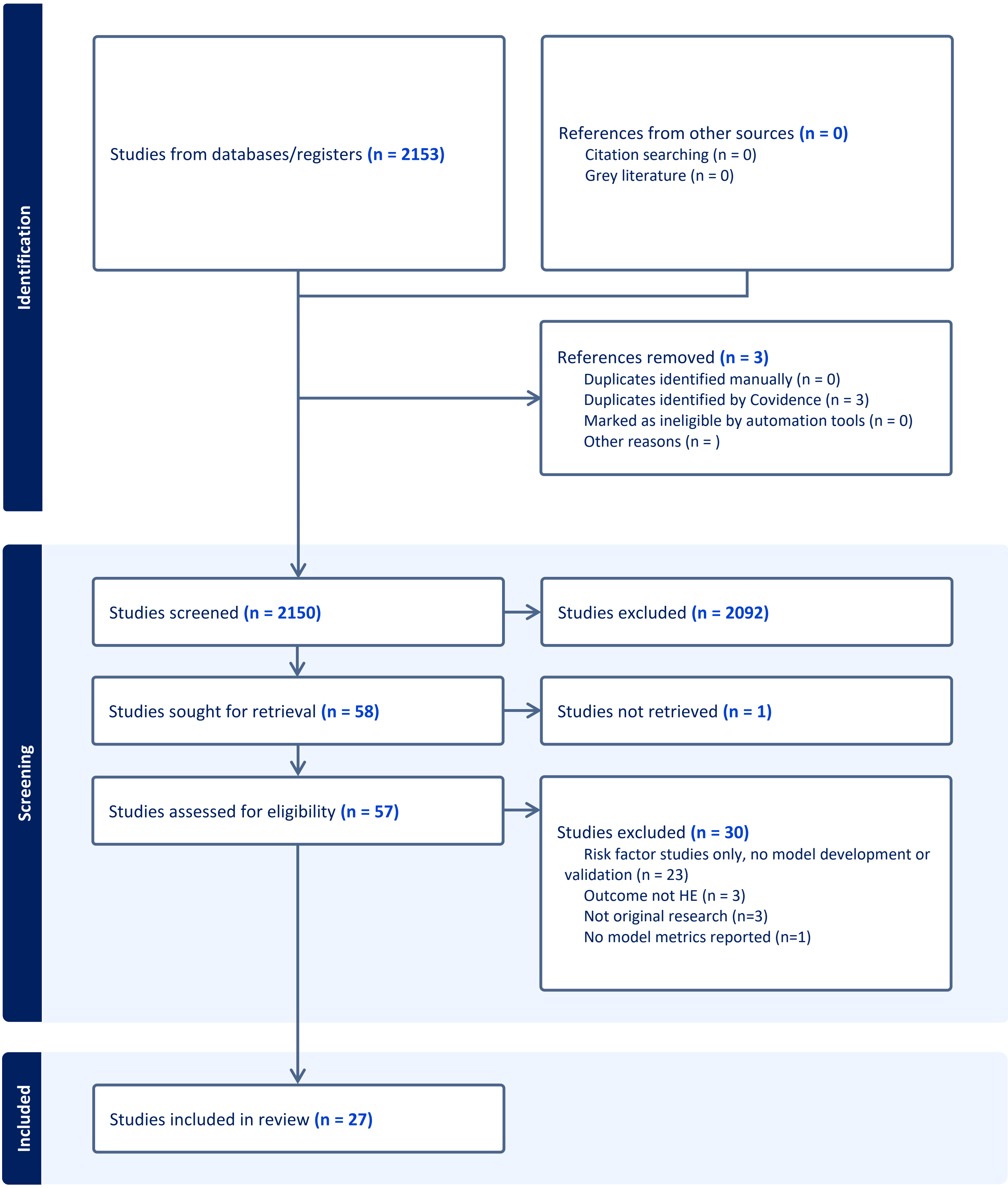

Fig. S2 Discrimination performance of established clinical scores across included studies

Study	Outcome (time window)	Participants/Events (N)	Estimate (range)
**Child-Pugh Score**			
Xu, 2025	severe HE (12-month)	406/78	0.70 (N.R.)
Liao, 2023	OHE (3-month)	207/39	0.66 (0.58–0.76)
Liu, 2021	OHE (NA)	159/37	0.66 (0.56–0.76)
Lin, 2021	HE (12-month)	224/82	0.65 (0.57–0.72)
Cai, 2022	HE (12-month)	241/83	0.64 (N.R.)
Jiang, 2025	OHE (12-month)	310/72	0.64 (0.51–0.76)
Wang, 2021	HE (12-month)	195/45	0.60 (0.51–0.68)
**MELD**			
Jiang, 2025	OHE (12-month)	310/72	0.75 (0.63–0.86)
Miao, 2025	OHE (12-month)	119/23	0.73 (0.51–0.93)
Xu, 2025	severe HE (1-month)	406/78	0.68 (N.R.)
Liao, 2023	OHE (3-month)	207/39	0.66 (0.56–0.76)
Lin, 2021	HE (12-month)	224/82	0.66 (0.58–0.73)
Cai, 2022	HE (12-month)	241/83	0.66 (N.R.)
Chen, 2025	Further decompensation (12 months)	152/52	0.65 (N.R.)
Wang, 2021	HE (12-month)	195/45	0.64 (0.55–0.72)
**MELD-Na**			
Chen, 2025	Further decompensation (12 months)	152/52	0.65 (N.R.)
**MELD 3.0**			
Chen, 2025	Further decompensation (12 months)	152/52	0.63 (N.R.)
**FIPS**			
Xu, 2025	severe HE (1-month)	406/78	0.78 (N.R.)
Cai, 2022	HE (12-month)	241/83	0.74 (0.68–0.80)
Xu, 2025	severe HE (12-month)	406/78	0.70 (N.R.)
**ALBI**			
Lin, 2021	HE (12-month)	224/82	0.74 (0.67–0.81)

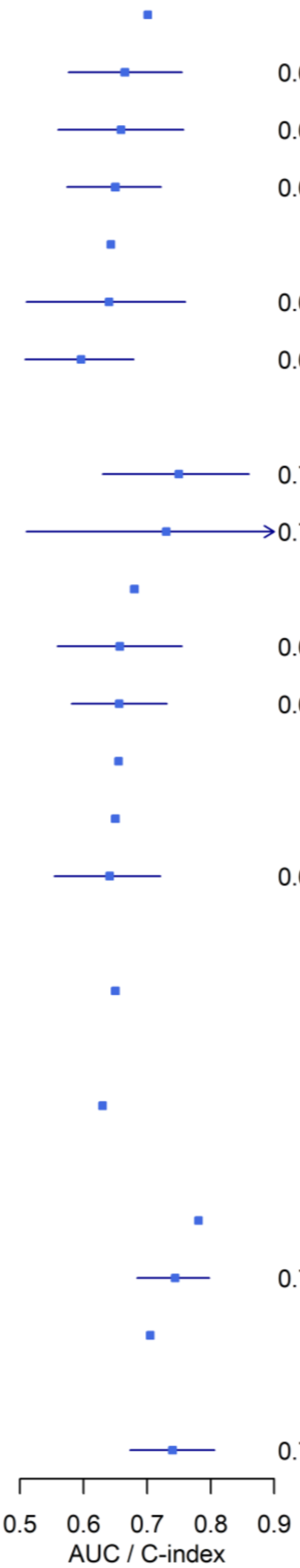

Supplement: Supplementary file 1 [file mmc1.pdf]
