# Supplementary material for: Risk prediction models for hepatic encephalopathy post-transjugular intrahepatic portosystemic shunt: A systematic review and critical appraisal
Source: JHEP Rep. 2026 Aug 22;8(11):102006. doi: 10.1016/j.jhepr.2026.102006 (PMC13694966; doi:10.1016/j.jhepr.2026.102006)

Research article

JHEP Reports

Risk prediction models for hepatic encephalopathy post-transjugular intrahepatic portosystemic shunt: A systematic review and critical appraisal

Authors

Stijn de Reus, Wenjing Wang, Wichor M. Bramer, …, Anna Baiges, Bettina E. Hansen, Raoel Maan

Correspondence

s.dereus.1@erasmusmc.nl (S. de Reus).

Graphical abstract

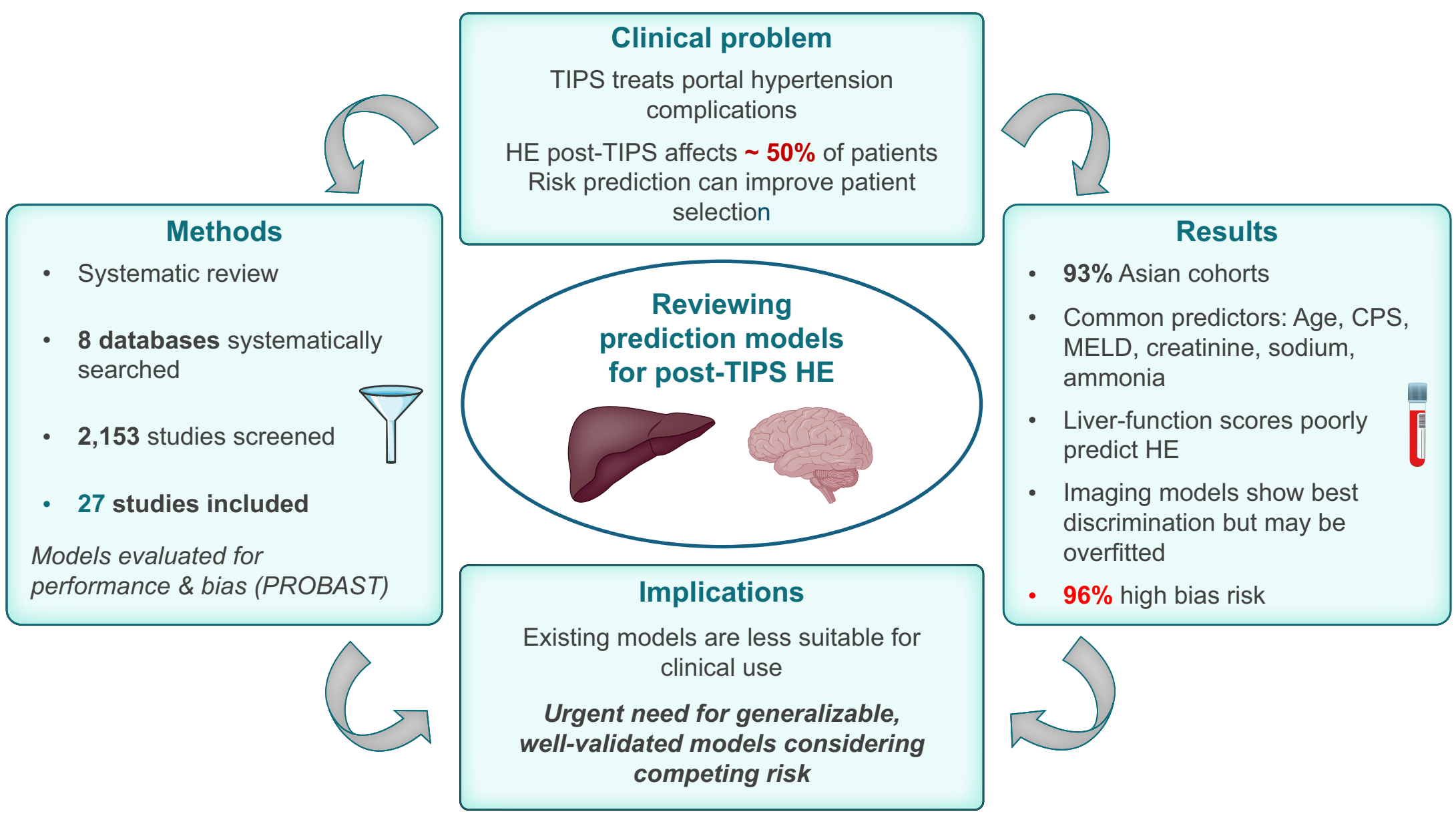

Highlights:

- Most prediction models are based on cirrhotic populations in Asian countries.
- Commonly used clinical predictors were age, Child-Pugh score, and creatinine.
- Imaging-based models perform best, but are hampered by low applicability.
- Main statistical flaws are model overfitting and not accounting for competing risk.
- Future models should account for clinical applicability and data complexities.

Impact and implications:

Despite numerous studies, there remains no consensus on whether prediction models for post-TIPS hepatic encephalopathy can be integrated into clinical practice. This paper provides a comprehensive overview of existing models by highlighting their strengths and limitations. In addition, it identifies recurrent predictors across models and proposes a framework for future research aimed at improving the prediction of this challenging complication.

https://doi.org/10.1016/j.jhepr.2026.102006

© 2026 The Author(s). Published by Elsevier B.V. on behalf of European Association for the Study of the Liver (EASL). This is an open access article under the CC BY license (http://creativecommons.org/licenses/by/4.0/). JHEP Reports, 2026, 8, 1–14

JHEP|Reports

Risk prediction models for hepatic encephalopathy post-transjugular intrahepatic portosystemic shunt: A systematic review and critical appraisal

Stijn de Reus[1,*,†], **Wenjing Wang**[2,†], Wichor M. Bramer[3], Juan-Carlos Garcia-Pagan[4], Anna Baiges[4], Bettina E. Hansen[2,5], Raoel Maan[1]

JHEP Reports **2026**. vol. 8 | 1–14

Check for updates

Background & Aims: Transjugular intrahepatic portosystemic shunt (TIPS) is an established treatment for portal hypertension (PH), with expanding indications. Hepatic encephalopathy (HE) is the main complication, affecting up to 50% of patients. Accurate prediction of HE risk is necessary for patient selection. Hence, we aimed to provide an overview of prediction models for post-TIPS HE.

Methods: We conducted a systematic review of studies developing or validating prediction models for post-TIPS HE that included ≥100 patients. The search, conducted up to December 2025, covered databases including Embase and MEDLINE. Outcomes assessed included AUCs/C-indices and risk of bias, evaluated using PROBAST.

Results: Twenty-seven studies were included, of which 24 developed prediction models. All used retrospective cohorts of patients with cirrhosis (population size: 106–621), with 25 cohorts from Asia (93%). Models varied in HE severity and prediction time frame, with 11 studies (41%) not reporting any. Discrimination varied across studies. The Child-Pugh and MELD scores, evaluated in nine studies, showed moderate performance (AUCs/C-indices: 0.60–0.75). Models that included imaging predictors (42%) reported higher discrimination than clinical models alone (0.73–0.97 *vs.* 0.61–0.86). Common clinical predictors were age (68%), Child-Pugh score (40%), and creatinine (28%). Models had a high risk of bias in PROBAST assessment (96%). Methodological concerns included inappropriate patient exclusion (63%), low events-per-variable ratio (81%), inadequate validation (79%), and neglect of competing risks (93%). Applicability concerns involved the use of highly selected populations (44%) and specialized imaging predictors (33%).

Conclusions: Current prediction models for post-TIPS HE, despite sometimes demonstrating strong discrimination, cannot reliably support clinical decision-making because of methodological and applicability concerns. Future research should prioritize models with accessible predictors, broad patient representation, appropriate consideration of competing risks, and rigorous validation to minimize overfitting.

© 2026 The Author(s). Published by Elsevier B.V. on behalf of European Association for the Study of the Liver (EASL). This is an open access article under the CC BY license (http://creativecommons.org/licenses/by/4.0/).

Introduction

Cirrhosis remains one of the leading causes of mortality worldwide.[1] Portal hypertensive (PH) complications, such as ascites, acute variceal bleeding (AVB), and hepatic encephalopathy (HE),[2] mark the state of decompensated cirrhosis. This reduces median survival of patients with cirrhosis to 2 years.[3] Transjugular intrahepatic portosystemic shunt (TIPS) is a well-known treatment for PH-related complications. Traditionally, TIPS was performed in the case of refractory ascites or for the treatment or prevention of AVB. Over recent years, TIPS indications have been reviewed and extended to situations such as prophylaxis of gastric varices[4] and prior to elective surgery to prevent post-surgical decompensation in selected cases.[5] Such expanding indications underline the increasing relevance of TIPS procedures in the treatment of PH. TIPS has strong long-term outcomes, showing improved survival in patients with refractory ascites[6] and AVB compared with standard of care.[7] The development of covered stent grafts for TIPS procedures is a possible contributor to this trend.[6,8] Therefore, TIPS currently provides treatment of PH regardless of transplant candidacy.[4]

Success rates of TIPS creation are high and often lead to resolution of PH-related complications; however, complications due to TIPS creation are common. HE is one of the main complications, resulting from reduced hepatic clearance of ammonia and other neurotoxins due to the created shunt.[9] The incidence of post-TIPS HE varies in clinical studies but can be as high as 53%.[10–12] Several studies have evaluated different strategies to prevent HE by giving medications prior to and after TIPS,[12–15] as well as how to better select participants.[16,17] There is no robust consensus on whether prophylactic

* Corresponding author. Address: Postbus 2040, 3000CA Rotterdam, the Netherlands. Tel.: 0107040704.
E-mail address: s.dereus.1@erasmusmc.nl (S. de Reus).
† Authors contributed equally as first authors.
https://doi.org/10.1016/j.jhepr.2026.102006

medications are effective in reducing HE risk;[18] hence, guidelines focus on careful patient selection.[4] In order to select patients appropriately, there is a need for prediction models to estimate the risk of HE.

Numerous predictors for post-TIPS HE have been suggested,[19] and research in this area is ongoing. Given the multifactorial nature of HE, individual predictors often fall short, making prediction models an area of increasing interest. A recent systematic review explored some of these models,[20] but it had a limited search strategy, focused on fewer databases, and concentrated on pooled model performance. It did not mention whether usable prediction models exist, nor did it highlight their clinical relevance. Our review builds on this by using a broader search strategy, refining inclusion criteria, increasing the minimum sample size of included studies, and providing a more comprehensive and robust assessment. We aim not only to summarize the models but also to appraise their clinical utility, determine whether a usable prediction model for post-TIPS HE exists and, if not, propose a new model strategy for further research.

Materials and methods

This systematic review was reported in accordance with the Preferred reporting items for systematic reviews and meta-analyses (PRISMA) Checklist[21] and the PRISMA-S extension to the PRISMA Statement for Reporting Literature Searches in Systematic Reviews.[22]

Data sources and study selection

The search strategy was developed by an experienced information specialist (WMB) with the lead authors (SR and WW). The search was developed in Embase.com, optimized for sensitivity and then translated to other databases following the method described by Bramer *et al.*[23] The search was carried out in the databases MEDLINE ALL via Ovid (1946 to Daily Update), Embase.com (1971-present), Web of Science Core Collection (Science Citation Index Expanded (1975-present); Social Sciences Citation Index (1975-present); Arts & Humanities Citation Index (1975-present); Conference Proceedings Citation Index- Science (1990-present); Conference Proceedings Citation Index- Social Science & Humanities (1990-present) and Emerging Sources Citation Index (2015-present)) and the Cumulative Index to Nursing and Allied Health Literature via EBSCOhost (1982-present). After the original search was performed on 01-07-2025, the search was last updated on 05-12-2025, using the methods described by Bramer *et al.*[24]

The search strategies for Medline, Embase and CINAHL used relevant thesaurus terms from Medical Subject Headings (MeSH), Emtree, and CINAHL headings, respectively. In all databases, additional terms were searched in titles, abstracts and author keywords. The search contained terms for 1) TIPS, 2) encephalopathy or survival, and 3) prediction, prognosis or artificial intelligence. Terms were combined with Boolean operators 'AND' and 'OR' and proximity operators were used to combine terms into phrases. The full search strategies for all databases are available in the supplementary materials [Supplementary Materials & methods; Table S1]. The searches were limited to exclude conference papers. Non-English articles were excluded from search results. We scanned retrieved non-included review articles for references, as well as articles citing these papers to scan for possible references missed by the search. No authors or subject experts were contacted and no extra unindexed journals in the field were browsed.

The references were imported into EndNote and duplicates were removed by the information specialist (WMB) using the method as described by Bramer *et al.*[25] Online software, Covidence, was used to perform a systematic review of identified studies. Two researchers (SR and WW) independently first screened titles and abstracts then full texts of candidate studies. Any discrepancies in decisions were resolved by discussion with a third researcher (BH).

We included studies developing and validating prediction models for post-TIPS HE. Papers that predicted HE as part of a composite endpoint were also eligible. We included adult patients with PH (regardless of the etiology of PH) for recognized indications of TIPS, as outlined by the EASL guidelines.[4] A minimum sample size of 100 patients was required to ensure an adequate number of HE events, based on HE occurrence in previous studies.[11,12] Studies that examined only causal associations or risk factors without developing a prediction model were excluded. Regarding study selection, there were no restrictions on which types of predictors were used in the model(s).

Data collection

Data collection was performed independently by two reviewers (WW and SR) based on the Critical Appraisal and Data Extraction for Systematic Reviews of Prediction Modelling Studies (CHARMS checklist).[26] Collected data included study design, outcome (severity of HE and time frame of prediction), sample size, underlying liver disease, indication for TIPS, stent type and size, method of predictor selection, model predictors and their timing (pre-, periprocedural- or post-TIPS). For AVB, we made a distinction between early and late TIPS. Early TIPS was defined as the use of TIPS in a salvage, rescue or preemptive setting, in compliance with guidelines.[4] It was not possible to subdivide the early TIPS group further, because this was not done in the studies. Late TIPS referred to secondary prophylaxis of recurrent variceal bleeding. Predictors were split into demographic and clinical predictors, history of PH-related complications, liver disease clinical scores, laboratory predictors, procedure-related predictors, imaging-based and body composition-based predictors. Procedure-related predictors encompassed all predictors assembled during the TIPS-procedure, be it prior to, during or after the actual TIPS creation. This decision was made to emulate everyday practice.

Quality assessment

Risk of bias and concerns regarding applicability were independently assessed by two reviewers (WW and SR) according to the Prediction Model Risk of Bias Assessment Tool (PROBAST).[27] Discrepancies were resolved through discussion with a third reviewer.

PROBAST is specific to prediction model studies and evaluates four domains: participants, predictors, outcome and analysis. In each domain, multiple signaling questions are used to identify potential sources of bias.[27] When one or more of these flaws are present, there is a high risk of bias, unless

sufficient justification is provided. Specific methodological questions include handling of missing data, methods of predictor selection, handling of competing risks, calibration methods, and whether and how internal/external validation was conducted.

To ensure consistency, predefined operational criteria were applied to selected PROBAST signaling questions. Studies were judged at high risk of bias in the participants domain if exclusions likely distorted the target population or outcome incidence, such as the exclusion of patients who died, underwent transplantation, were lost to follow-up after TIPS (>10% or unreported), had substantial missing data (>20% or unreported), or if early TIPS was excluded despite the model being intended for all TIPS procedures. In the predictors domain, variables not available prior to TIPS (*e.g.* portal pressure gradient or periprocedural complications) were considered high risk when the model aimed at pre-procedural prediction. In the analysis domain, <10 events per variable (EPV) for model development and <100 events for external validation in regression models was considered to be high risk for overfitting. Machine learning models were assessed on a case-by-case basis, considering sample size, model complexity, and use of penalization. Complete-case analysis was considered high risk when ≥20% of data were missing. Regarding applicability, highly selected populations (*e.g.* single-etiology cohorts or transplant waitlist patients) and non-routinely available predictors raised concerns.

Results

Characteristics of studies

The article search and selection process is illustrated in the PRISMA flow diagram (Fig. S1). After removing duplicates, 2,096 of 2,153 articles were excluded based on title and abstract. After full-text review, 27 studies were included. The primary reason for exclusion was that the authors did not develop a prediction model (23/30, 77%).

Of the included studies, 24 developed prediction models,[28–51] and three studies performed external validation of established liver disease scores[52–54] (Table 1). All studies were among patients with cirrhosis, with two studies also including patients with non-cirrhotic portal hypertension (NCPH) in their analysis.[34,39] Sample size ranged from 106 to 621 patients and 25/27 (93%) were comprised of Asian populations.[29-33,35-54] In many studies using Asian cohorts (84%), overlap of participants could not be ruled out. Among the 24 original model studies, five reported external validation using independent datasets, with sample sizes ranging from 24 to 208 participants.[29,30,33,42,48]

Nearly all studies included AVB as an indication for TIPS (25/27, 93%),[29-42,44-54] with four choosing to exclude early TIPS (16%)[29,30,47,54] and two looking at early TIPS specifically.[49,51] Refractory ascites was included in 23 studies (85%),[28-40,42-48,52-54] including two studies restricted to ascites only.[28,43] Other indications included refractory hepatic hydrothorax,[45,54] portal vein thrombosis,[39] Budd-Chiari syndrome,[34] and hepatorenal syndrome.[54] Five studies included “other” indications without further specification,[34,35,38,45,53] and one study included “preventative” treatment, without mention of what TIPS was intended to prevent.[39]

Regarding the best-performing original prediction models of each study, all models included pre-procedural predictors, with five models (21%) also including peri-procedural predictors[31,34,38,45,48] (Fig. 1). No model included post-TIPS predictors.

Clinical outcomes in studies

Outcome definitions and prediction time horizons varied across studies. Overt HE was the most common outcome predicted in 63% (17/27) of the studies,[29-33,35,37,38,40-42,44,45,47-51] followed by any HE grade (26%).[34,36,39,43,46,52,54] Two studies predicted severe HE (8%).[28,53] Of all studies, two examined HE as part of a composite endpoint.[28,31] Only 16 studies specified a prediction time frame;[28,31,34-39,42,43,48,50-54] of which 12 months was the most commonly used (10/16, 63%).[28,31,35,37,39,42,50,52–54] This means that 11 studies did not specify a prediction time frame for HE (41%).[29,30,32,33,40,41,44–47,49] In total, 11 studies excluded patients with prior HE (41%). Three of these excluded any history of HE regardless of grade.[40,42,45] Three studies excluded patients with prior overt HE only,[30,48,51] while two others excluded patients with “preoperative” HE without specifying the relevant time window, leaving it unclear whether this referred to HE at index admission or any prior episode.[38,54] Three studies excluded patients with a history of severe or grade III–IV HE.[32,39,43]

Prior HE was reported in eight studies (33% among studies that did not exclude HE),[28,34,35,41,43,50,51,53] with incidence ranging from 4–41% in studies evaluating any prior HE,[34,35,41,43] compared with 4–7% for previous overt HE,[28,50,53] and 3% for covert HE.[51] In studies using overt HE as the endpoint, 11–33% of patients developed HE after TIPS,[29-33,35,37,38,40-42,44,45,47-51] whereas models predicting any HE reported incidences of 23–48%,[34,36,39,43,46,52,54] and those for severe HE reported incidences of 19–26%[28,53] (Table 1). The study by Xu *et al.* defined severe HE as HE requiring hospitalization and reported that 19% of patients were readmitted with grade III–IV HE after the index admission.[53] Other studies did not distinguish HE-related hospitalization as a separate outcome.

Model performance

Clinical models

Nine studies evaluated whether established liver disease scores, determined prior to TIPS, could predict post-TIPS HE[31,35,38-40,42,52-54] (Fig. S2). Across these studies, with differing HE severity and prediction time frame, Child-Pugh score (CPS) had a range of area under the curves/concordance indices (AUCs/C-indices) of 0.60–0.70 (corresponding CI-width 0.51–0.76), and model for end-stage liver disease (MELD) score showed a similar pattern with a range of 0.64–0.75 (0.51–0.93). The FIPS (Freiburg index of Post-TIPS survival) showed a range of 0.70–0.78 (0.68–0.78),[52,53] while albumin-bilirubin index (ALBI), tested in only one study, showed an AUC of 0.74 (0.67–0.81).[39]

Discriminatory performances of original clinical prediction models varied greatly, with the lowest AUC being 0.61 (0.51–0.70)[32] and the highest being 0.86 (CI not reported)[51] (Fig. 2). Considering all the tested models,[28-30,32-39,41,45,47-51]

Table 1. Characteristics of prediction models studies for the risk of post-TIPS hepatic encephalopathy.

Study	Model[1]	Etiology of PH	Indication	Centers	Outcome (time horizon)	No. of sample/ events[2]	Predictor timing	Modelling method	Performance[3] (AUC/C-index, 95% CI)
**Model development studies (n = 24)**									
Aicart -Ramos, 2021	Simple Nomogram	Cirrhosis	Ascites	Multi-center	Mortality or recurrent severe HE[4] (12-month)	159/63	pre-TIPS	FGR	0.70 (N.R.)
Chen, 2023	ModelC-3D	Cirrhosis	Variceal bleeding (early TIPS excluded), ascites	Multi-center	Overt HE	390/101	pre-TIPS	SVM	0.82 (N.R.)
Chen, 2024	ModelC-V	Cirrhosis	Variceal bleeding (early TIPS excluded), ascites	Multi-center	Overt HE	413/131	pre-TIPS	LR	0.81 (N.R.)
Chen, 2025	/	Cirrhosis	Variceal bleeding, ascites	Single center	Further decompensation[5] (12-month)	152/52	pre- and during TIPS	LR	0.784 (N.R.)
Cheng, 2022	Radiomics model	Cirrhosis	Variceal bleeding, ascites	Single center	Overt HE	106/24	pre- and during TIPS	RF	0.89 (0.76, 1.0)
Cheng, 2024	Radiomic + clinical LRM	Cirrhosis	Variceal bleeding, ascites	Single center	Overt HE	85/19	pre-TIPS	LR	0.83 (0.69, 0.92)
Ince, 2023	LRM	Cirrhosis, NCPH	Variceal bleeding, ascites, BCS, other	Single center	Any HE (1-month)	327/105	pre- and during TIPS	LR	0.83 (0.82, 0.84)
Jiang, 2025	/	Cirrhosis	Variceal bleeding, ascites, other	Single center	Overt HE (12-month)	310/72	pre-TIPS	LR	0.86 (0.78, 0.94)
Li, 2019	/	Cirrhosis	Variceal bleeding, ascites	Single center	Any HE (3-month)	183/51	pre-TIPS	LR	0.82 (0.72, 0.91)
Li, 2024	/	Cirrhosis	Variceal bleeding, ascites	Single center	Overt HE (12-month)	92/27	pre-TIPS	Cox	0.75 (0.60, 0.90)
Liao, 2023	Nomogram	Cirrhosis	Variceal bleeding, ascites, other	Single center	Overt HE (3-month)	207/39	pre- and during TIPS	LR	0.85 (0.74, 0.95)
Lin, 2021	ALBI-age model	Cirrhosis, NCPH	Variceal bleeding, ascites, thrombosis, preventive treatment	Single center	Any HE (12-month)	224/82	Pre-TIPS	Cox	0,7 (N.R.)
Liu, 2021	CP-SVol grading system	Cirrhosis	Variceal bleeding, ascites	Single center	Overt HE	159/37	pre-TIPS	Cox	N.R.
Liu, 2025	LRM	Cirrhosis	Variceal bleeding	Single center	Overt HE	152/23	pre- TIPS, during TIPS	LR	0.83 (N.R.)
Miao, 2025[6]	Combined TabPFN model	Cirrhosis	Variceal bleeding, ascites	Multi-center	Overt HE (12-month)	119/23	pre-TIPS	TabPFN	0.90
Mukund, 2026	/	Cirrhosis	Ascites	Single center	Any HE (6-month)	282/77	pre-TIPS	LR	N.R.
Shi, 2025	ScoreALL	Cirrhosis	Variceal bleeding, ascites	Multi-center	Overt HE	571/187	pre-TIPS	LR	N.R.
Tong, 2021	/	Cirrhosis	Variceal bleeding, ascites, hydrothorax, other	Single center	Overt HE	299/52	pre- and during TIPS	LR	0.78 (0.72, 0.85)
Wang, 2023	Male/female model	Cirrhosis	Variceal bleeding, ascites	Single center	Any HE	Male: 116/56 Female 75/33	pre-TIPS	LR	Male: 0.81 (0.74, 0.89) Female: 0.80 (0.71, 0.90)

(*continued on next page*)

Table 1. (*continued*)

Study	Model[1]	Etiology of PH	Indication	Centers	Outcome (time horizon)	No. of sample/ events[2]	Predictor timing	Modelling method	Performance[3] (AUC/C-index, 95% CI)
Yang, 2021	Model CI	Cirrhosis	Variceal bleeding (early TIPS excluded), ascites	Multi-center	Overt HE	130/N.R.	pre-TIPS	LR	0,97 (N.R.)
Yang, 2022a	Full model	Cirrhosis	Variceal bleeding, ascites	Multi-center	Overt HE (3 months)	276/61	pre- and during TIPS	LR	0.77 (0.71, 0.83)
Yang, 2022b	Model.CR	Cirrhosis	Early TIPS	Multi-center	Overt HE	127/36	pre-TIPS	LR	0.90 (N.R.)
Yin, 2020	/	Cirrhosis	Variceal bleeding	Single center	Overt HE (12-month)	264/83	pre-TIPS	Cox	0.78 (N.R.)
Zhong, 2021	ANN model	Cirrhosis	Early TIPS	Single center	Overt HE (3-month)	207/33	pre-TIPS	ANN	0.86 (N.R.)
**External validation studies (n = 3)**									
Cai, 2022	FIPS score	Cirrhosis	Variceal bleeding, ascites	Single center	Any HE (12-month)	241/83	pre-TIPS	Existing score	0.74 (0.68, 0.80)
Xu, 2025[6]	FIPS score	Cirrhosis	Variceal bleeding, ascites, other	Single center	Severe HE[4] (1-month)	406/78	pre-TIPS	Existing score	0.78 (N.R.)
	CLIFC-AD score				Severe HE[4] (12-month)				0.70 (N.R.)
Wang, 2021	Child-Pugh score	Cirrhosis	Variceal bleeding (urgent TIPS excluded), ascites, hydrothorax, HRS	Single center	Any HE (12-month)	195/45	pre-TIPS	Existing score	0,60 (0.51, 0.68)
	MELD score								0.64 (0.55, 0.72)

ANN, artificial neural network; AUC, area under the receiver operating characteristic curve; BCS, Budd–Chiari syndrome; C-index, concordance index; CLIF-AD, Chronic Liver Failure – Acute Decompensation; FGR, Fine–Gray regression; FIPS, Freiburg index of Post-TIPS survival; HE, hepatic encephalopathy; HRS, Hepatorenal syndrome; LRM, logistic regression model; NCPH, non-cirrhotic portal hypertension; N.R., not reported; PH, portal hypertension; RF, random forest; SVM, support vector machine; TabPFN, Tabular Prior-data Fitted Network; TIPS, transjugular intrahepatic portosystemic shunt.

[1]If multiple models were reported in a study, the best-performing model was presented.

[2]Event numbers for the training cohort were not explicitly reported in certain articles and were derived from the available reported data.

[3]If internal validation performance was not available, apparent (training cohort) performance was reported.

[4]Severe HE was defined as grade III–IV (West Haven criteria).

[5]Further decompensation was defined as: 1) A second PH-driven decompensation (ascites, AVB, or HE); or 2) Recurrent variceal bleeding, worsening of ascites, recurrent HE, spontaneous bacterial peritonitis or hepatorenal syndrome.

[6]Model reported multiple time-dependent AUC's; the highest AUC is shown.

shown in Fig. 2, the most frequently included predictors were age (68%), CPS (40%), creatinine (28%), MELD(-Na) (20%), sodium (16%), and ammonia (12%). When looking specifically at the best-performing prediction model of each study (Fig. 1), this trend held for aforementioned predictors, with age being included in 82% of the best-performing models. Frequently tested predictors which ended up being excluded from models were etiology of liver disease (included in 2/5 studies), albumin, activated partial thromboplastin time, prothrombin time, and international normalized ratio (not included in 2-4 studies in which they were tested) (Fig. 1). Regarding specific models, Zhong *et al.* predicted overt HE three months post-TIPS using age >65 years, ALBI, biological sex, alcohol-related cirrhosis and MELD; with a notable C-index of 0.86 (CI not reported).[51] Jiang *et al.* created a preoperative nomogram which included age, prognostic nutritional index, prior HE, ammonia and creatinine. It also reached an AUC of 0.86 (0.78–0.94) to predict overt HE at 12 months.[35]

In terms of prediction time frame, models forecasting HE one month after TIPS had an AUC range of 0.82–0.83 (0.82–0.84).[34] For models predicting at 3 months, the AUC/C-indices ranged from 0.68–0.86 (0.61–0.95),[36,38,48,51] while those predicting at 12 months showed a range of 0.66–0.86 (0.56–0.94)[28,35,37,39,50] (Fig. 2). AUCs/C-indices were different across prediction time frames. Notably, 80% of models predicting HE at 3 months had an AUC ≥0.80, compared to only 13% of models at 12 months.

Imaging-based models

Seven models, originating from six studies, used only imaging-based predictors.[29,30,32,33,47,49] AUCs ranged from 0.73–0.97 (CI not reported) (Fig. 2). 63% of models had AUCs >0.80 for predicting overt HE.[32,33,47,49] Six of the models looked at vasculature-derived parameters. Common predictors of such models were the diameter ratio of portal *vs*. splenic vein(s) (PSR, 50% of models) and (maximal) diameter of the hepatic fissure (HFMD, 33%) (Fig. 1). The latest study by Cheng *et al.* looked at radiomic features of visceral adipose tissue instead of hepatic vasculature.[33] The paper by Yang *et al.* reported an AUC of 0.97 (CI not reported) with HFMD and PSR as predictors.[47]

In nine studies, models combined clinical and imaging predictors.[29-31,33,42,46-49] AUCs/C-indices ranged from 0.73–0.97 (CI range not reported), with the majority of models having discriminatory performance >0.80 (73%).[29,30,33,42,47,49] When looking at Fig. 1, only one of the previously mentioned seven imaging models retained a higher AUC when using a model containing only imaging-based predictors instead of a combined model.[32]

Yang *et al.* replicated an AUC of 0.97 (CI not reported) with a combined model using aforementioned predictors as well as bilirubin and CPS.[47] Miao *et al.* combined signatures from four different models based on radiomics of specific regions (liver, spleen, abdominal fat and abdominal muscle) and MELD, to predict overt HE at 12 months, with an AUC of 0.90 (CI not reported).[42]

Risk of bias and concerns regarding applicability

Of all studies, 17 (63%) scored a high risk of bias in the participants domain due to inappropriate exclusion of patients[29-33,38,39,41,43-47,49,51-53] (Fig. 3). This included exclusion of patients lost to follow-up, those who died early or underwent liver transplantation (LT), those with low albumin levels, as well as exclusion based on post-TIPS stent dysfunction or revision. Regarding participants, there were concerns regarding applicability in 12 of the studies (44%).[28-30,33,39,41,42,47,49-52] Six studies (50%) had narrow inclusion criteria, with low generalizability.[29,30,33,39,47,52] Six studies (50%) looked at subsets of specific TIPS indications.[28,41,42,49–51]

Nearly all studies had a low risk of bias in the PROBAST predictor domain (96%). Often, accessible demographic, clinical or laboratory predictors were used, and their timing was correct. The study by Cheng *et al.* scored a high risk of bias because they used periprocedural portal pressure gradient in a pre-operative model, contrary to our timing definition of predictors.[32] Ten studies raised applicability concerns (37%).[29,32-34,40,44,46-49] Seven of these used predictors based on CT-image derivatives that require additional software or radiological expertise (70%).[29,40,44,46–49] Two studies used radiomics, which is also reliant on additional software.[32,33] The study by Ince *et al.* included the operator as a predictor, which is center-specific.[34]

Most studies had a low risk of bias in the outcome domain (56%). Risk of bias in 41% of the studies was determined to be "unsure", due to authors not using a prediction time frame, meaning that the time between predictor assessment and outcome was unclear.[29,30,32,33,40,41,44–47,49] Given that the risk of bias would probably depend on the length of follow-up in these studies, which was frequently omitted, no stronger assumption about the risk of bias was made. The study by Mukund *et al.* had a high risk of bias due to insufficient reporting of the grade of HE predicted and how the outcome (HE) was defined.[43] The concern regarding applicability was also graded as "unsure" in studies lacking a prediction time frame.

All but one study (96%) scored a high risk of bias in the analysis domain due to one or more methodological weaknesses[48] (Table 2). Fifteen studies (56%) did not detail how they dealt with missing data,[28-30,32,36,39,42,43,45,47,49,51-54] and eight studies (30%) handled missing data inappropriately (*e.g*. performing a complete case analysis without reporting the number of missing data).[33,35,37,38,40,41,44,46] Competing risks were unaccounted for in 25 studies (93%), except two studies by Aicart-Ramos *et al.* and Yang *et al.* that performed a Fine-Gray analysis.[28,48] Multiple factors could have contributed to possible overfitting, including low EPV, with 22 models (81%) falling below recommended thresholds (EPV ≥10 for model development and ≥100 events for external validation).[28-30,32,33,36-42,45-54] Furthermore, 17 studies (71%) used univariable screening as part of their predictor selection strategy.[28,30,31,33,36-40,42-47,50,51] Internal validation was frequently absent (33%) or performed by random split-sample (46%), with cross-validation and bootstrapping infrequently used (21%).[31,32,34,41,48] Finally, although model discrimination metrics were commonly reported; nine studies (33%) did not calibrate their prediction model(s).[33,34,36,40,43–45,51,54]

Discussion

Despite the growing interest in prediction models for post-TIPS HE, this review finds several important challenges. Most

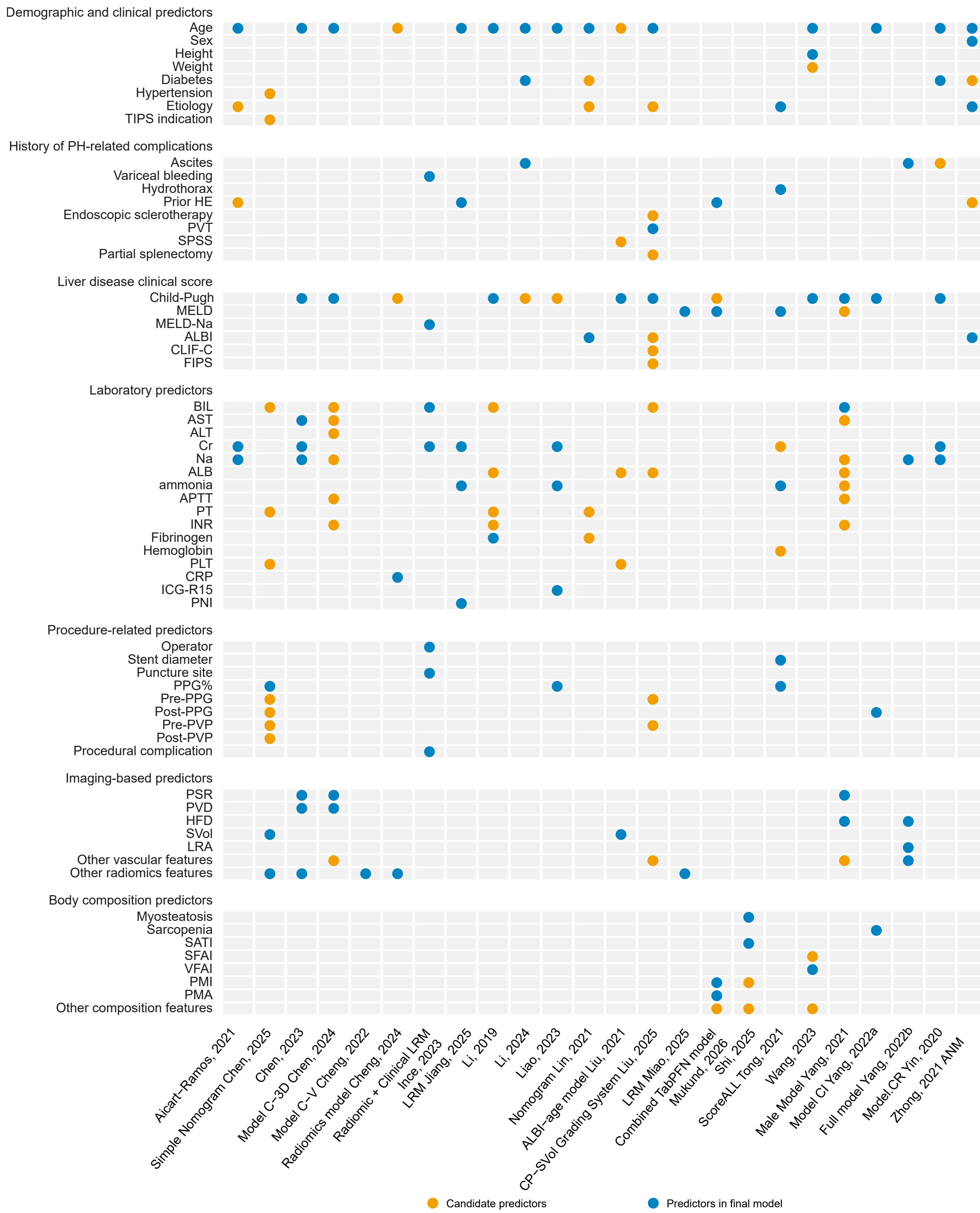

Fig. 1. Heatmap of candidate and final predictors used in the models. Each cell corresponds to whether a given predictor was evaluated in a specific model. Yellow dots indicate predictors that were considered in multivariable analyses but were not retained in the final model (candidate predictors). Blue dots represent predictors that were retained in the final model. In total, 24 studies comprising 25 models are included. If multiple models were reported in a study, only the model identified by the authors as the best-performing one, or the model with the highest AUC/C-index, was presented in this figure. Liu, 2021 reported separate models for male and female patients, and both models are shown. ALB, albumin; ALBI, albumin–bilirubin score; ALT, alanine aminotransferase; APTT, activated partial thromboplastin time; AST, aspartate aminotransferase; BIL, bilirubin; BUN, blood urea nitrogen; CLIF-C, Chronic Liver Failure Consortium; Cr, Creatinine; CRP, C-reactive protein; FIPS, Freiburg index of Post-TIPS survival; HFD, hepatic fissure diameter; HSD, splenic vein diameter; INR, international normalized ratio; LLA, left lobe anteroposterior diameter; LRA, right lobe anteroposterior diameter; MELD, model for end-stage liver disease; MHD, middle hepatic vein diameter; PAB, pre-albumin; PH, portal hypertension; PMA, psoas muscle area; PMI, psoas muscle index; PSR, portal-to-splenic vein ratio; PLT, platelet; PNI, prognostic nutritional index (10 × albumin (g/dl)+0.005 × total lymphocyte count (/mm^3)); PPG, portal pressure gradient; PSR, portal-to-splenic vein ratio; PT, prothrombin time; PVD, portal vein diameter; PVP, portal vein pressure; PVT, portal vein thrombosis; SATI, subcutaneous adipose tissue index; SFAI, subcutaneous fat area index; SPSS, spontaneous portosystemic shunt; SVol, spleen volume; VFAI, visceral fat area index; TIPS, transjugular intrahepatic portosystemic shunt.

Model type/study/model	Outcome (time window)	Participants/events (N)	Estimate (range)
**Clinical model**			
**Zhong, 2021**			
ANN model	Overt HE (3 months)	207/33	0.86 (N.R.)
LRM			0.82 (N.R.)
**Jiang, 2025**			
	Overt HE (12 months)	310/72	0.86 (0.78–0.94)
**Liao, 2023**			
Nomogram	Overt HE (3 months)	207/39	0.85 (0.74–0.95)
**Yang, 2021**			
ModelC	Overt HE (N.R.)	130/N.R.	0.83 (N.R.)
**Ince, 2023**			
LRM	Any HE (1 month)	327/105	0.83 (0.82–0.84)
Cat−Boost model			0.83 (0.82–0.83)
SVM			0.82 (0.82–0.83)
**Liu, 2025**			
LRM	Overt HE (N.R.)	152/23	0.82 (N.R.)
XG−Boost model			0.80 (N.R.)
RF model			0.70 (N.R.)
**Li, 2019**			
	Any HE (3 months)	183/51	0.82 (0.72–0.91)
**Tong, 2021**			
	Overt HE (N.R.)	299/52	0.78 (0.72–0.85)
**Yin, 2020**			
	Overt HE (12 months)	264/83	0.78 (N.R.)
**Cheng, 2024***			
Clinical LRM	Overt HE (N.R.)	85/19	0.76 (0.55–0.88)
**Chen, 2024***			
ModelC	Overt HE (N.R.)	413/131	0.75 (N.R.)
**Li, 2024**			
	Overt HE (12 months)	92/27	0.75 (0.6–0.9)
**Yang, 2022b**			
Model.C	Overt HE (N.R.)	127/36	0.72 (N.R.)
**Chen, 2023***			
ModelC	Overt HE (N.R.)	390/101	0.72 (N.R.)
**Aicart−Ramos, 2021**			
Nomogram	Mortality or recurrent severe HE (12 months)	159/63	0.70 (N.R.)
Age + MELD			0.69 (N.R.)
Age + MELD−Na			0.69 (N.R.)
**Lin, 2021**			
ALBI model	Any HE (12 months)	224/82	0.70 (N.R.)
**Yang, 2022a***			
Core model	Overt HE (3 months)	276/61	0.68 (0.61–0.75)
**Cheng, 2022**			
Clinical model	Overt HE (N.R.)	106/24	0.61 (0.511–0.701)
**Image model**			
**Yang, 2021**			
ModelI	Overt HE (N.R.)	130/N.R.	0.97 (N.R.)
**Cheng, 2022**			
Radiomics model	Overt HE (N.R.)	106/24	0.89 (0.76–1)
**Yang, 2022b**			
Model.R	Overt HE (N.R.)	127/36	0.86 (N.R.)
**Cheng, 2024***			
Radiomic LRM	Overt HE (N.R.)	85/19	0.81 (0.68–0.91)
**Chen, 2023***			
Model3D	Overt HE (N.R.)	390/101	0.73 (N.R.)
Model2D			0.62 (N.R.)
**Chen, 2024***			
ModelV	Overt HE (N.R.)	413/131	0.73 (N.R.)
**Combined model**			
**Yang, 2021**			
ModelCI	Overt HE (N.R.)	130/N.R.	0.97 (N.R.)
**Yang, 2022b**			
Model.CR	Overt HE (N.R.)	127/36	0.90 (N.R.)
**Miao, 2025***			
Combined TabPFN model	Overt HE (12 months)	119/23	0.90 (N.R.)
**Cheng, 2024***			
Radiomic + clinical LRM	Overt HE (N.R.)	85/19	0.83 (0.69–0.92)
**Chen, 2023***			
ModelC−3D	Overt HE (N.R.)	390/101	0.82 (N.R.)
**Chen, 2024***			
ModelC−V	Overt HE (N.R.)	413/131	0.81 (N.R.)
**Wang, 2023**			
Male model	Any HE (N.R.)	116/56	0.81 (0.74–0.89)
Female model		75/33	0.80 (0.71–0.9)
**Chen, 2025**			
	Further decompensation (12 months)	152/52	0.78 (N.R.)
**Yang, 2022a***			
Full model	Overt HE (3 months)	276/61	0.77 (0.71–0.83)
Sarcopenia model			0.73 (0.66–0.8)

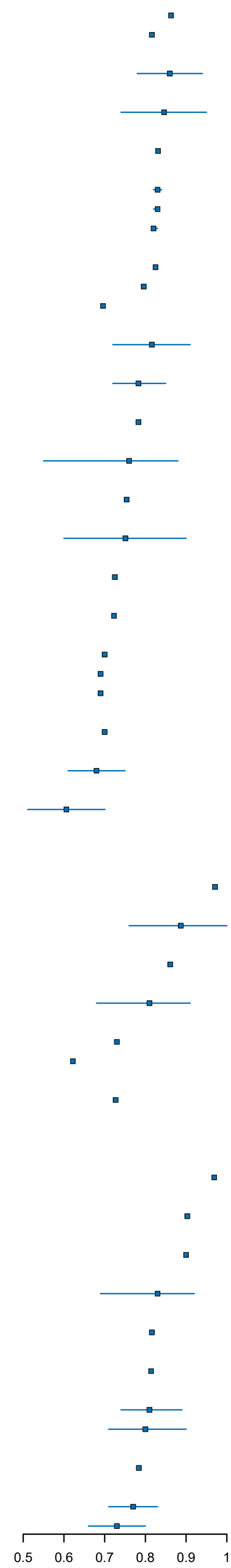

Fig. 2. Discrimination performance of prediction models for post-TIPS HE. Models are grouped by HE definition and follow-up duration. Points represent reported AUC or C-index values. Horizontal lines represent 95% CIs when available. *Models marked with an asterisk underwent external validation. ALBI, albumin–bilirubin; ANN, artificial neural network; AUC, area under the receiver operating characteristic curve; C-index, concordance index; HE, hepatic encephalopathy; LRM, logistic regression model; N.R., not reported; RF, random forest; SVM, support vector machine; TIPS, transjugular intrahepatic portosystemic shunt; XG-Boost, extreme gradient boosting.

Table 2. Assessment of methodological quality of the prediction models.

Study	Model name	Missing data	Competing risk	Predictors selection	Calibration	Internal validation	Clinical tool	External validation	
								No. of sample/ events	Performance (AUC/C-index, 95% CI)
Aicart-Ramos, 2021	Nomogram	N.R.	Yes	Univariable, clinical relevance	Calibration plot	N.R.	Nomogram	/	/
Chen, 2023	ModelC-3D	N.R.	No	LASSO	Isotonic regression, Calibration plot	N.R.	Web-based applet	97 (51)	0.816 (N.R.)
Chen, 2024	ModelC-V	N.R.	No	Univariable, LASSO	Calibration plot	N.R.	Web-based applet	208 (57)	0.781 (N.R.)
Chen, 2025	/	MI	No	Univariable, LASSO	HL test, Calibration plot	Bootstrap	Nomogram	/	/
Cheng, 2022	Radiomics model	N.R.	No	RF	Calibration plot	CV	None	/	/
Cheng, 2024	Radiomic + clinical LRM	CCA	No	Univariable, LASSO	N.R.	Random split sample,	None	24 (7)	0.84 (0.72, 0.92)
Ince, 2023	LRM	CCA	No	SFS	N.R.	CV	None	/	/
Jiang, 2025	/	CCA	No	LASSO	Calibration plot	Random split sample	Nomogram	/	/
Li, 2019	/	N.R.	No	Univariable, stepwise	N.R.	Random split sample	None	/	/
Li, 2024	/	CCA	No	Univariable	Calibration plot	Random split sample	Nomogram	/	/
Liao, 2023	/	CCA	No	Univariable	Calibration plot, Bootstrap calibration	Random split sample	Nomogram	/	/
Lin, 2021	ALBI model	N.R.	No	Univariable, clinical relevance	Calibration plot	N.R.	Nomogram	/	/
Liu, 2021	CP-SVol Grading System	CCA	No	Univariable	N.R.	N.R.	Grading system	/	/
Liu, 2025	LRM	CCA	No	Embedded methods, RFE	Calibration plot, Brier score	CV	None	/	/
Miao, 2025	Combined TabPFN	N.R.	No	Univariable, LASSO	Calibration plot	Random split sample	None	47 (12)	0.91 (N.R.)
Mukund, 2026	/	N.R.	No	Univariable	N.R.	N.R.	None	/	/
Shi, 2025	ScoreALL	CCA	No	Univariable	N.R.	N.R.	Risk score	/	/
Tong, 2021	/	N.R.	No	Univariable	N.R.	Random split sample	Risk score	/	/
Wang, 2023	Male model/female model	CCA	No	Univariable	Calibration plot	N.R.	Nomogram	/	/
Yang, 2021	ModelCI	N.R.	No	Univariable	Calibration plot	Random split sample	Nomogram	/	/
Yang, 2022a	Full model	MI	Yes	Clinical relevance, LASSO	HL test, Brier score, Calibration plot	Bootstrap	Nomogram	182 (33)	0.78 (0.68, 0.86)

(*continued on next page*)

Table 2. (*continued*)

Study	Model name	Missing data	Competing risk	Predictors selection	Calibration	Internal validation	Clinical tool	External validation: No. of sample/ events	External validation: Performance (AUC/C-index, 95% CI)
Yang, 2022b	Model.CR	N.R.	No	Stepwise, LASSO	HL test, Calibration plot	Random split sample	Nomogram	/	/
Yin, 2020	/	CCA	No	Univariable	Calibration plot	Random split sample	Nomogram	/	/
Zhong, 2021	ANN model	N.R.	No	Univariable	N.R.	Random split sample	None	/	/

This table summarizes all original multivariable prediction models reported in each study.
AUC, area under the receiver operating characteristic curve; ALBI, albumin-bilirubin score; ANN, artificial neural network; CCA, complete case analysis; C-index, concordance index; CV, cross-validation; HL, Hosmer–Lemeshow test; LASSO, least absolute shrinkage and selection operator; LRM, logistic regression model; MELD, model for end-stage liver disease; MI, multiple imputation; RF, random forest; RFE, recursive feature elimination; N.R., not reported; RF, random forest; SFS, sequential feature selection; SVM, support vector machine; TabPFN, Tabular Prior-data Fitted Network; XG-Boost, extreme gradient boosting.

notably, the majority of studies focused on Asian cohorts, limiting generalizability. In addition, although imaging-based and combined models offer superior performance, statistical limitations greatly hinder their application. As a result, no current model provides reliable, real-world utility for clinical decision-making. Nevertheless, the existing literature provides a strong foundation for determining the prognostic information physicians seek in pre- and post-TIPS care.

All but two studies focused on Asian cohorts. This restricts model usage in broader populations because the burden of cirrhosis is different in Asian countries, where chronic hepatitis B and C remain prevalent. While the burden of metabolic dysfunction-associated steatotic liver disease is similar between Asia, Europe and North America, that of alcohol-related cirrhosis is lower in Asia.[1] Given that etiology was included in two final models,[45,51] this warrants attention.

Age, CPS, creatinine, MELD, sodium, and ammonia, the latter typically measured prior to or at the time of TIPS, consistently emerged as key variables in prediction models. This suggests that they are promising targets for developing a reliable prediction model and is in line with a previous meta-analysis looking at important predictors of post-TIPS HE.[19] This is also supported by the EASL guidelines, which advise selection based partly on CPS and MELD.[4] However, application of established liver disease scores alone led to relatively poor discrimination, which is logical given that they were not built for HE prediction. When comparing clinical and imaging-based models, discrimination was highest in the latter and in combined models. There is evidence to support the use of imaging predictors. Body composition measurements are supported by previous evidence on the effect of sarcopenia on HE risk.[55] A relation between muscle mass and HE has been suggested, with lower muscle mass leading to reduced ammonia clearance by glutamine synthetase.[56] Another study investigated using magnetic resonance imaging features as outcome predictors in cirrhosis and provides an example of why liver-specific morphological assessment could possibly predict HE.[57] While this evidence could explain the better performance of imaging-based models, these models frequently employed machine-learning techniques,[29,32,42] which generally require more data to achieve stable performance. For example, one study has shown that random forest methodology may require more than 20-fold events per predictor when compared with logistic regression.[58] Without enough events, the apparent superiority of imaging-based models is likely overoptimistic, reflecting overfitting rather than true predictive gain.

We identified possible methodological concerns in the prediction models, particularly the omission of a prediction time frame, in 41% of studies. From a clinical perspective, it seems logical that HE that occurs long after TIPS placement is less likely to be related to the creation of a shunt. Hence, we were concerned that inclusion of HE events far removed from shunt creation could dilute or skew analyses. Furthermore, the time of patient follow-up was not mentioned in such studies, so it is possible that individual patients within a study may have had varying follow-up times, which could also impact conclusions. This was mainly the case for imaging-based models; *e.g.* Model^{I} by Yang *et al.*[47] and Radiomics model by Cheng *et al.*[32] With regard to clinical models, there seemed to be a trend of shorter prediction time frames being associated with

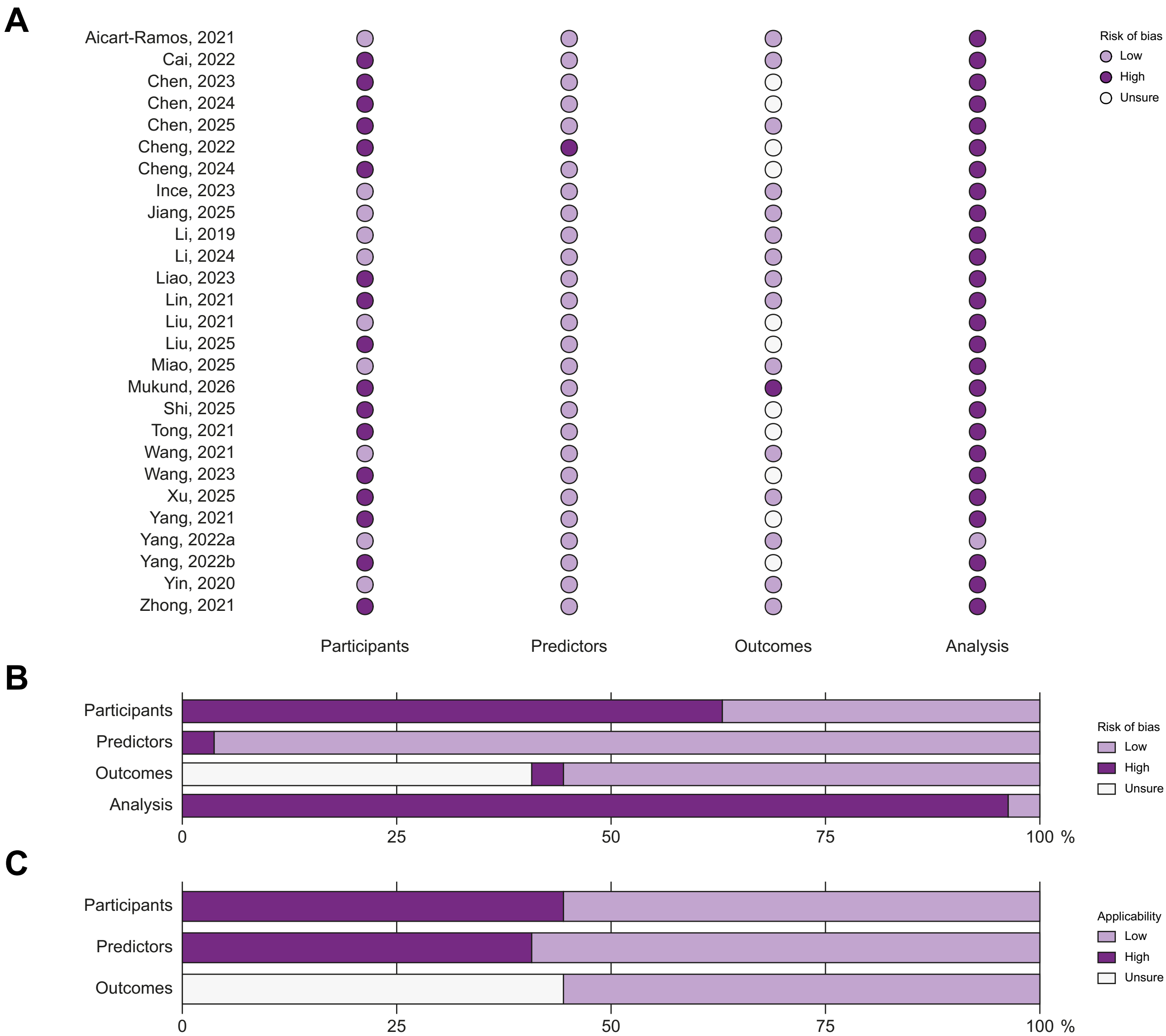

Fig. 3. Assessment of risk of bias and applicability using the PROBAST tool. (A) Risk of bias assessment across four PROBAST domains (participants, predictors, outcomes, and analysis) for each included study. (B) Proportion of studies rated as low, high, or unclear risk of bias for each domain. (C) Proportion of studies rated as low, high, or unclear concern for applicability across three domains.

stronger discrimination. Evidence supports this as post-TIPS HE mainly occurs in the first months after TIPS, with one multi-center retrospective study of 377 post-TIPS HE cases describing a median time to occurrence of 26 days.[59] Furthermore, prediction models generally perform better in the short-term.[60] This is not to say that there are no applications for longer prediction frames, such as predicting recurrent HE or the need for stent reduction, but they should be made explicit by researchers. In the analyses, the competing risks of death and LT were unaccounted for in most studies, which could have led to biased effect estimates. Well-performing clinical models like those of Jiang *et al.*[35] and Zhong *et al.*[51] omitted competing risks. The two models that did account for this (Aicart-Ramos *et al.*,[28] Yang *et al.*[48]) did not achieve an AUC/C-index >0.80. This raises the question of whether other models overestimated performance by oversimplifying complex TIPS cohorts. Moreover, calibration was frequently omitted (33%) and models were rarely validated using resampling techniques (21%). This is concerning, given that calibration is more sensitive than discrimination to overfitting, small sample sizes, and high-dimensional predictors.[61] Together, these methodological flaws limit model robustness and hinder the translation of existing models into routine clinical practice. Given that these methodological flaws extend beyond the analytical domain, they limit the overall strength of the evidence provided by the included studies.

The primary limitation of our study was the inability to perform a meta-analysis due to significant heterogeneity across studies, especially the reporting of outcomes (AUC *vs.* C-index) and omission of standard errors. We determined that further analyses would not enhance our findings and could risk drawing incorrect conclusions. In contrast to the approach

taken in the previous systematic review,[20] we believe such attempts can distort the evidence. Furthermore, the evidence our study provides is hampered by the lack of well-defined endpoints for HE outcomes. Only one study, by Xu *et al.*, used HE-related hospitalization in its outcome definition.[53] Hence, other studies were dependent on research physicians interpreting the severity of HE retrospectively. Additionally, the omission of a history of HE in 67% of studies is concerning, as it implies that known risk factors for post-TIPS HE[4] were not properly characterized. Regarding our bias assessment, we recognized that PROBAST's strict criteria, particularly in TIPS-related studies, posed challenges. Certain requirements, such as a standard EPV, are difficult to meet in these populations. While PROBAST effectively assesses the external validity of prediction models across different TIPS cohorts, we still found valuable insights in studies that scored poorly, particularly the identification of key predictors. A point of concern remains that we found possible overlap in patient populations in multiple studies.[29,30,32,33,36,38-42,44-49,51-54] We accepted this risk, given the qualitative nature of our review and the fact that models often tested different predictors. While cases could have overlapped, they were ultimately incorporated in distinct ways due to the differing approaches of the models. Additionally, we included studies that also looked at NCPH, given that the majority of their cohorts were composed of patients with cirrhosis. For the two studies in question, patients with NCPH comprised 5% and 12% of the total study population, respectively.[34,39] We are aware that our conclusions do not extend to this population. In comparing our work with the previously published systematic review,[20] we identified notable differences, especially in our search strategy. This allowed us to have a more comprehensive study selection.

This systematic review could not identify a prediction model suitable for the whole TIPS population because of the aforementioned shortcomings. Given the evidence, however, we can deduce that a prediction model for post-TIPS HE should predict HE in the short term, with a well-defined prediction time frame. It should be applicable and not rely on complicated software or machine-learning techniques. Perhaps the ideal model is a combination of imaging and clinical predictors, with imaging predictors being easily assessed and not hampering TIPS planning. Calibration and resampling techniques should be used to validate the model, and for it to hold in external validation. While we acknowledge the need for a model that is broadly generalizable, we recognize that specific subgroups may also require tailored risk assessment, and the development of models in these populations should not be discouraged. With TIPS gaining a more prominent role in the treatment of PH, further research into such models should be conducted to support clinical decision-making in this vulnerable patient population.

Affiliations

[1]*Department of Gastroenterology and Hepatology, Erasmus MC, Rotterdam, the Netherlands;* [2]*Department of Epidemiology, Erasmus MC, Rotterdam, the Netherlands;* [3]*Medical Library, Erasmus MC University Medical Center, Rotterdam, the Netherlands;* [4]*Barcelona Hepatic Hemodynamic Laboratory, Liver Unit, Hospital Clínic, Clínic Barcelona, ERN-RareLiver (Health Care Provider of the European Reference Network on Rare Liver Disorders), CSUR (Centro de referencia del Sistema Nacional de Salud en Enfermedad Hepática Vascular Compleja en adultos), FRCB-IDIBAPS (Fundació de Recerca Clínic Barcelona-Institut d'Investigacions Biomèdiques August Pi i Sunyer), CIBEREHD (Centro de Investigación Biomédica en Red Enfermedades Hepáticas y Digestivas), AGAUR SGR202101115, Medicine Department, Faculty of Medicine, and health Science, Universitat de Barcelona, Barcelona, Spain;* [5]*IHPME, University of Toronto & Toronto Centre for Liver Disease, University Health Network, Toronto, Canada*

Abbreviations

AUC, area under the curve; ALBI, albumin-bilirubin; C-index, concordance index; CPS, Child-Pugh score; EASL, European Association for the Study of the Liver; EPV, events per variable; HE, hepatic encephalopathy; LT, liver transplantation; MELD, model for end-stage liver disease; NCPH, non-cirrhotic portal hypertension; PH, portal hypertension; PROBAST, Prediction model Risk Of Bias Assessment Tool; TIPS, transjugular intrahepatic portosystemic shunt.

Financial support

Authors did not receive financial support for the creation of this paper.

Conflicts of interest

SR, WW, WMB and AB declared no conflicts of interest. JCGP received grants/research support from Instituto de Salud Carlos III, Cook Medical, and Gore Medical. JCGP was a consultant or advisor for AstraZeneca, Cook Medical, Gore Medical, and GSK. BH received grants/research support from Ipsen, Mirum, Advanz, and Gilead. BH was an advisor for Ipsen, Mirum, Advanz, Gilead, Falk, and Alfasigma. RM received speaker's fees from Gore Medical, Chiesi, Janssen-Cilag, and AbbVie.

Please refer to the accompanying ICMJE disclosure forms for further details.

Authors' contributions

SR, WW, BH and RM were involved in all aspects of the paper. WMB was involved in methodology and writing. JCGP and AB were involved in writing of the paper.

Data availability

All data analyzed in this study are derived from previously published studies, which are cited in the manuscript. Summaries of the extracted data are available from the corresponding author upon reasonable request.

Acknowledgements

We would like to thank our colleagues for contributing to the article through informal discussions and brainstorming. Secondly, we would like to shine a light on researchers who have investigated this topic in order to improve TIPS-care for patients worldwide.

Supplementary data

Supplementary data to this article can be found online at https://doi.org/10.1016/j.jhepr.2026.102006.

References

Author names in bold designate shared co-first authorship

[1] **Zhang Y**, **Luo M**, Ming Y. Global burden of cirrhosis and other chronic liver diseases caused by specific etiologies from 1990 to 2021. BMC Gastroenterol 2025;25(1):641.

[2] **Engelmann C**, **Clària J**, **Szabo G**, et al. Pathophysiology of decompensated cirrhosis: portal hypertension, circulatory dysfunction,

inflammation, metabolism and mitochondrial dysfunction. J Hepatol 2021;75(Suppl 1):S49–S66. Suppl 1.
[3] European Association for the Study of the L. EASL Clinical Practice Guidelines for the management of patients with decompensated cirrhosis. J Hepatol 2018;69(2):406–460.
[4] European Association for the Study of the L. EASL clinical practice guidelines on TIPS. J Hepatol 2025;83(1):177–210.
[5] Manzano-Nunez R, Jimenez-Masip A, Chica-Yanten J, et al. Unlocking the potential of TIPS placement as a bridge to elective and emergency surgery in cirrhotic patients: a meta-analysis and future directions for endovascular resuscitation in acute care surgery. World J Emerg Surg 2023;18(1):30.
[6] Bureau C, Thabut D, Oberti F, et al. Transjugular intrahepatic portosystemic shunts with covered stents Increase transplant-Free survival of patients with cirrhosis and recurrent ascites. Gastroenterology 2017;152(1): 157–163.
[7] Larrue H, D'Amico G, Olivas P, et al. TIPS prevents further decompensation and improves survival in patients with cirrhosis and portal hypertension in an individual patient data meta-analysis. J Hepatol 2023;79(3):692–703.
[8] Perello MP, Mur JP, Vives MS, et al. Long-term follow-up of transjugular intrahepatic portosystemic shunt (TIPS) with stent-graft. Diagn Interv Radiol 2019;25(5):346–352.
[9] Friis KH, Thomsen KL, Laleman W, et al. Post-transjugular intrahepatic portosystemic shunt (TIPS) hepatic encephalopathy-A review of the Past Decade's literature focusing on incidence, risk factors, and prophylaxis. J Clin Med 2023;13(1).
[10] Fonio P, Discalzi A, Calandri M, et al. Incidence of hepatic encephalopathy after transjugular intrahepatic portosystemic shunt (TIPS) according to its severity and temporal grading classification. Radiol Med 2017;122(9): 713–721.
[11] Casadaban LC, Parvinian A, Minocha J, et al. Clearing the Confusion over hepatic encephalopathy after TIPS creation: incidence, prognostic factors, and clinical outcomes. Dig Dis Sci 2015;60(4):1059–1066.
[12] Bureau C, Thabut D, Jezequel C, et al. The Use of rifaximin in the prevention of overt hepatic encephalopathy after transjugular intrahepatic portosystemic shunt : a randomized controlled trial. Ann Intern Med 2021;174(5): 633–640.
[13] Riggio O, Masini A, Efrati C, et al. Pharmacological prophylaxis of hepatic encephalopathy after transjugular intrahepatic portosystemic shunt: a randomized controlled study. J Hepatol 2005;42(5):674–679.
[14] Riggio O, Nardelli S, Pasquale C, et al. No effect of albumin infusion on the prevention of hepatic encephalopathy after transjugular intrahepatic portosystemic shunt. Metab Brain Dis 2016;31(6):1275–1281.
[15] de Wit K, Schaapman JJ, Nevens F, et al. Prevention of hepatic encephalopathy by administration of rifaximin and lactulose in patients with liver cirrhosis undergoing placement of a transjugular intrahepatic portosystemic shunt (TIPS): a multicentre randomised, double blind, placebo controlled trial (PEARL trial). BMJ Open Gastroenterol 2020;7(1).
[16] **Lv Y**, **Zuo L**, **Zhu X**, et al. Identifying optimal candidates for early TIPS among patients with cirrhosis and acute variceal bleeding: a multicentre observational study. Gut 2019;68(7):1297–1310.
[17] Lv Y, Bai W, Zhu X, et al. Development and validation of a prognostic score to identify the optimal candidate for preemptive TIPS in patients with cirrhosis and acute variceal bleeding. Hepatology 2024;79(1):118–134.
[18] European Association for the Study of the L. EASL Clinical Practice Guidelines on the management of hepatic encephalopathy. J Hepatol 2022;77(3):807–824.
[19] Bai M, Qi X, Yang Z, et al. Predictors of hepatic encephalopathy after transjugular intrahepatic portosystemic shunt in cirrhotic patients: a systematic review. J Gastroenterol Hepatol 2011;26(6):943–951.
[20] **Fang Z**, **Liu Z**, Pu Z, et al. Risk prediction models for hepatic encephalopathy following TIPS: a systematic review and meta-analysis. Front Med (Lausanne) 2026;13:1707035.
[21] Moher D, Liberati A, Tetzlaff J, et al. Preferred reporting items for systematic reviews and meta-analyses: the PRISMA statement. J Clin Epidemiol 2009;62(10):1006–1012.
[22] Rethlefsen ML, Kirtley S, Waffenschmidt S, et al. PRISMA-S: an extension to the PRISMA statement for reporting literature searches in systematic reviews. Syst Rev 2021;10(1):39.
[23] Bramer WM, de Jonge GB, Rethlefsen ML, et al. A systematic approach to searching: an efficient and complete method to develop literature searches. J Med Libr Assoc 2018;106(4):531–541.
[24] Bramer W, Bain P. Updating search strategies for systematic reviews using EndNote. J Med Libr Assoc 2017;105(3):285–289.
[25] Bramer WM, Giustini D, de Jonge GB, et al. De-duplication of database search results for systematic reviews in EndNote. J Med Libr Assoc 2016;104(3):240–243.
[26] **Moons KG**, **de Groot JA**, Bouwmeester W, et al. Critical appraisal and data extraction for systematic reviews of prediction modelling studies: the CHARMS checklist. Plos Med 2014;11(10):e1001744.
[27] **Wolff RF**, **Moons KGM**, Riley RD, et al. PROBAST: a tool to assess the risk of bias and applicability of prediction model studies. Ann Intern Med 2019;170(1):51–58.
[28] **Aicart-Ramos M**, **Ibáñez-Samaniego L**, Hernández-Gea V, et al. A simple nomogram identifies patients with refractory ascites with worse outcome after transjugular intrahepatic portosystemic shunt. Eur J Gastroenterol Hepatol 2021;33(1S Suppl 1):e587–e593.
[29] **Chen X**, **Wang T**, **Ji Z**, et al. 3D automatic liver and spleen assessment in predicting overt hepatic encephalopathy before TIPS: a multi-center study. Hepatol Int 2023;17(6):1545–1556.
[30] **Chen X**, **Huang M**, **Yu X**, et al. Hepatic-associated vascular morphological assessment to predict overt hepatic encephalopathy before TIPS: a multi-center study. Hepatol Int 2024;18(4):1238–1248.
[31] **Chen X**, **Lin Y**, **Jia K**, et al. Hepatosplenic volumes and portal pressure gradient identify one-year further decompensation risk post-transjugular intrahepatic portosystemic shunt. J Clin Translational Hepatol 2025;13(10): 835–846.
[32] Cheng S, Yu X, Chen X, et al. CT-based radiomics model for preoperative prediction of hepatic encephalopathy after transjugular intrahepatic portosystemic shunt. Br J Radiol 2022;95(1132):20210792.
[33] **Cheng S**, **Hu G**, Jin Z, et al. Prediction of hepatic encephalopathy after transjugular intrahepatic portosystemic shunt based on CT radiomic features of visceral adipose tissue. Acad Radiol 2024;31(5):1849–1861.
[34] İnce O, Önder H, Gençtürk M, et al. Machine learning insights: predicting hepatic encephalopathy after TIPS placement. Cardiovasc Intervent Radiol 2023;46(12):1715–1725.
[35] Jiang Z, Chu J, Han Z, et al. Development and validation of a preoperative clinical parameter-based nomogram to predict overt hepatic encephalopathy within 1 year after transjugular intrahepatic portosystemic shunt. Front Med (Lausanne) 2025;12:1634368.
[36] Li Y, He X, Pang H. A model to predict early hepatic encephalopathy in patients undergoing transjugular intrahepatic portosystemic shunt. Turk J Gastroenterol 2019;30(8):702–707.
[37] Li K, Cheng Y, Zhao R, et al. Prediction of mortality and overt hepatic encephalopathy undergoing transjugular intrahepatic portosystemic shunt: a retrospective cohort study. Abdom Radiol (Ny) 2024;49(3):908–918.
[38] **Liao Y**, **Zhang L**, **Wang JT**, et al. A novel nomogram predicting overt hepatic encephalopathy after transjugular intrahepatic portosystemic shunt in portal hypertension patients. Sci Rep 2023;13(1):15244.
[39] Lin X, Gao F, Wu X, et al. Efficacy of albumin-bilirubin score to predict hepatic encephalopathy in patients underwent transjugular intrahepatic portosystemic shunt. Eur J Gastroenterol Hepatol 2021;33(6):862–871.
[40] **Liu J**, **Zhou C**, Wang Y, et al. The combination of Child-Pugh score and quantitative CT-based spleen volume could predict the risk of hepatic encephalopathy after transjugular intrahepatic portosystemic shunt creation. Abdom Radiol (Ny) 2021;46(7):3464–3470.
[41] **Liu DJ**, **Jia LX**, Zeng FX, et al. Machine learning prediction of hepatic encephalopathy for long-term survival after transjugular intrahepatic portosystemic shunt in acute variceal bleeding. World J Gastroenterol 2025;31(4):100401.
[42] **Miao L**, **Zhao H**, Zhang X, et al. Enhancing post-TIPS hepatic encephalopathy risk stratification: a hybrid TabPFN model leveraging radiomics, deep transfer learning features, and MELD score. Hepatol Int 2025;20(2):428–440.
[43] **Mukund A**, **Bhardwaj V**, Jindal A, et al. Transjugular intrahepatic portosystemic shunt related hepatic encephalopathy in cirrhotics with refractory ascites: incidence and Correlation with TIPS stent diameter and pre-TIPS sarcopenia. J Clin Exp Hepatol 2026;16(1):103125.
[44] **Shi W**, **Xu W**, **Fan N**, et al. Body compositions Correlate with overt hepatic encephalopathy after transjugular intrahepatic portosystemic shunt: a multicentre cohort study. J Clin Gastroenterol 2025;59(3):262–268.
[45] **Tong H**, **Gan C**, Wei B, et al. Risk factors for overt hepatic encephalopathy after transjugular intrahepatic portosystemic shunt creation in patients with liver cirrhosis. J Dig Dis 2021;22(1):31–40.
[46] **Wang C**, **Teng Y**, **Gao J**, et al. Low adipose tissue index as an indicator of hepatic encephalopathy in cirrhotic patients following transjugular intrahepatic portosystemic shunt. Abdom Radiol (Ny) 2023;48(4):1454–1467.

[47] Yang Y, Fu S, Cao B, et al. Prediction of overt hepatic encephalopathy after transjugular intrahepatic portosystemic shunt treatment: a cohort study. Hepatol Int 2021;15(3):730–740.

[48] Yang C, Zhu X, Liu J, et al. Development and validation of prognostic models to estimate the risk of overt hepatic encephalopathy after TIPS creation: a multicenter study. Clin Transl Gastroenterol 2022;13(3):e00461.

[49] Yang Y, Liang X, Yang S, et al. Preoperative prediction of overt hepatic encephalopathy caused by transjugular intrahepatic portosystemic shunt. Eur J Radiol 2022;154:110384.

[50] Yin X, Zhang F, Guo H, et al. A nomogram to predict the risk of hepatic encephalopathy after transjugular intrahepatic portosystemic shunt in Cirrhotic Patients. Sci Rep 2020;10(1):9381.

[51] Zhong BY, Wang WS, Shen J, et al. Single-centre retrospective training cohort using artificial Intelligence for prognostic prediction of encephalopathy, mortality, and liver dysfunction after early TIPS creation. Cardiovasc Intervent Radiol 2021;44(10):1597–1608.

[52] Cai W, Zheng B, Lin X, et al. Prediction of patient hepatic encephalopathy risk with Freiburg index of post-TIPS survival score following transjugular intrahepatic portosystemic shunts: a retrospective study. Int J Gen Med 2022;15:4007–4016.

[53] Xu XJ, Yin L, Zhu YJ, et al. Comparing efficacy of different scoring models to predict hepatic encephalopathy after TIPS in cirrhotic patients. Ann Med 2025;57(1):2514082.

[54] Wang Z, Wu YF, Yue ZD, et al. Comparative study of indocyanine green-R15, Child-Pugh score, and model for end-stage liver disease score for prediction of hepatic encephalopathy after transjugular intrahepatic portosystemic shunt. World J Gastroenterol 2021;27(5):416–427.

[55] Nardelli S, Lattanzi B, Torrisi S, et al. Sarcopenia is risk factor for development of hepatic encephalopathy after transjugular intrahepatic portosystemic shunt placement. Clin Gastroenterol Hepatol 2017;15(6):934–936.

[56] Nardelli S, Lattanzi B, Merli M, et al. Muscle Alterations are associated with minimal and overt hepatic encephalopathy in patients with liver cirrhosis. Hepatology 2019;70(5):1704–1713.

[57] Bradley CR, Cox EF, Scott RA, et al. Multi-organ assessment of compensated cirrhosis patients using quantitative magnetic resonance imaging. J Hepatol 2018;69(5):1015–1024.

[58] Silvey S, Liu J. Sample size requirements for popular classification Algorithms in Tabular clinical data: Empirical study. J Med Internet Res 2024;26:e60231.

[59] Kabelitz MA, Sandmann L, Praktiknjo M, et al. Early occurrence of hepatic encephalopathy following transjugular intrahepatic portosystemic shunt Insertion is Linked to Impaired survival: a multicenter cohort study. Clin Gastroenterol Hepatol 2025;23(12):2214–2223 e27.

[60] Bansal A, Heagerty PJ. A Tutorial on evaluating the time-varying discrimination Accuracy of survival models used in Dynamic decision making. Med Decis Making 2018;38(8):904–916.

[61] Van Calster B, McLernon DJ, van Smeden M, et al. Calibration: the Achilles heel of predictive analytics. BMC Med 2019;17(1):230.

Keywords: Liver cirrhosis; Idiopathic noncirrhotic portal hypertension; Risk factors; Radiology; Interventional; Ascites; Hemorrhage; Bias (risk).

Received 13 May 2026; received in revised form 13 August 2026; accepted 18 August 2026; Available online 22 August 2026

Journal of Hepatology, Volume *8*

Supplemental information

Risk prediction models for hepatic encephalopathy post-transjugular intrahepatic portosystemic shunt: A systematic review and critical appraisal

Stijn de Reus, Wenjing Wang, Wichor M. Bramer, Juan-Carlos Garcia-Pagan, Anna Baiges, Bettina E. Hansen, and Raoel Maan

Risk prediction models for hepatic encephalopathy post-transjugular intrahepatic portosystemic shunt: A systematic review and critical appraisal

Stijn de Reus, Wenjing Wang, Wichor M. Bramer, Juan-Carlos Garcia-Pagan, Anna Baiges, Bettina E Hansen, Raoel Maan

Table of contents

Table S1……………………………………………………………………………………2

Fig. S1…………………………………………………………………………………...5

Fig. S2…………………………………………………………………………………...6

Table S1. Search terms used for systematic review

Database searched	Platform	Years of coverage	Records	Records after duplicates removed
Medline ALL	Ovid	1946 - Present	1039	1031
Embase	Embase.com	1971 - Present	1462	645
Web of Science Core Collection*	Web of Knowledge	1975 - Present	1210	345
CINAHL Plus	EBSCO	1982 - Present	334	123
**Total**			**4045**	**2144**

*Science Citation Index Expanded (1975-present) ; Social Sciences Citation Index (1975-present) ; Arts & Humanities Citation Index (1975-present) ; Conference Proceedings Citation Index- Science (1990-present) ; Conference Proceedings Citation Index- Social Science & Humanities (1990-present) ; Emerging Sources Citation Index (2005-present)

No other database limits were used than those specified in the search strategies

Search terms

**Medline**
(Portasystemic Shunt, Transjugular Intrahepatic / OR (((transjug* OR trans-jug*) ADJ3 (intrahepat* OR intra-hepat* OR portosyst* OR port*-syst*) ADJ6 shunt*) OR post-tips OR posttips OR ptips OR post-tipss OR posttipss OR ptipss).ab,ti,kf. OR (tips OR tipss).ti.) AND (Brain Diseases / OR Hepatic Encephalopathy/ OR exp Survival / OR exp Mortality / OR (encephalopath* OR hepatocereb* OR hepatoencephalopath* OR surviv* OR mortalit*).ab,ti,kf. OR (he OR ohe).ti.) AND (Prediction Algorithms/ OR Prediction Methods, Machine/ OR Prognosis/ OR Risk/ OR Risk Factors/ OR Validation Study/ OR Nomograms/ OR exp Machine Learning/ OR Machine Learning Algorithms/ OR exp Artificial

Intelligence/ OR (predict* OR prognos* OR Risk-score* OR Risk-factor* OR risk-model* OR validation* OR nomogra* OR ((machine OR deep) ADJ learning) OR (artificial* ADJ3 intelligen*)).ab,ti,kf. OR (risk OR index OR model*).ti.) NOT (news OR congres* OR abstract* OR book* OR chapter* OR dissertation abstract*).pt. NOT (exp animals/ NOT humans/) AND english.la.
**Embase**
('transjugular intrahepatic portosystemic shunt'/de OR 'TIPS endoprosthesis'/de OR (((transjug* OR trans-jug*) NEAR/3 (intrahepat* OR intra-hepat* OR portosyst* OR port*-syst*) NEAR/6 shunt*) OR post-tips OR posttips OR ptips OR post-tipss OR posttipss OR ptipss):ab,ti,kw OR (tips OR tipss):ti) AND (encephalopathy/exp OR survival/exp OR mortality/exp OR 'mortality risk'/de OR (encephalopath* OR hepatocereb* OR hepatoencephalopath* OR surviv* OR mortalit*):ab,ti,kw OR (he OR ohe):ti) AND (prediction/exp OR prognosis/de OR risk/de OR 'risk factor'/de OR 'risk score model'/de OR 'risk score'/de OR 'risk scoring system'/de OR 'risk model'/de OR 'mortality risk'/de OR 'validation process'/de OR 'validation study'/de OR nomogram/de OR 'machine learning'/exp OR 'artificial intelligence'/exp OR (predict* OR prognos* OR Risk-score* OR Risk-factor* OR risk-model* OR validation* OR nomogra* OR ((machine OR deep) NEXT/1 learning) OR (artificial* NEAR/3 intelligen*)):ab,ti,kw OR (risk OR index OR model*):ti) NOT ([Conference Abstract]/lim OR [Conference Review]/lim OR [preprint]/lim OR 'clinical trial':it) NOT ([letter]/lim OR [editorial]/lim) NOT ([animals]/lim NOT [humans]/lim) AND [english]/lim
**Web of science**
(TS=(((transjug* OR trans-jug*) NEAR/2 (intrahepat* OR intra-hepat* OR portosyst* OR port*-syst*) NEAR/5 shunt*) OR post-tips OR posttips OR ptips OR post-tipss OR posttipss OR ptipss) OR TI=(tips OR tipss)) AND (TS=(encephalopath* OR hepatocereb* OR hepatoencephalopath* OR surviv* OR mortalit*) OR TI=(he OR ohe)) AND (TS=(predict* OR prognos* OR Risk-score* OR Risk-factor* OR risk-model* OR validation* OR nomogra* OR ((machine OR deep) NEAR/1 learning) OR (artificial* NEAR/2 intelligen*)) OR TI=(risk OR index OR model*)) NOT DT=(Meeting Abstract OR Meeting Summary) AND LA=(English)

CINAHL
(XB(((transjug* OR trans-jug*) N2 (intrahepat* OR intra-hepat* OR portosyst* OR port*-syst*) N5 shunt*) OR post-tips OR posttips OR ptips OR post-tipss OR posttipss OR ptipss) OR TI(tips OR tipss)) AND (MH Brain Diseases OR MH Hepatic Encephalopathy+ OR MH Survival + OR MH Mortality + OR XB(encephalopath* OR hepatocereb* OR hepatoencephalopath* OR surviv* OR mortalit*) OR TI(he OR ohe)) AND (MH Prediction Algorithms+ OR MH Prediction Algorithms + OR MH Prediction Models OR MH Predictive Validity OR MH Prognosis+ OR MH Risk+ OR MH Risk Factors+ OR MH Validation Studies + OR MH Machine Learning+ OR MH Machine Learning Algorithms+ OR MH Artificial Intelligence+ OR XB(predict* OR prognos* OR Risk-score* OR Risk-factor* OR risk-model* OR validation* OR nomogra* OR ((machine OR deep) N1 learning) OR (artificial* N2 intelligen*)) OR TI(risk OR index OR model*)) NOT PT(news OR congres* OR abstract* OR book* OR chapter* OR dissertation abstract*) NOT (MH animals+ NOT MH humans) AND LA(english)

Fig. S1 PRISMA 2020 flow diagram of study selection

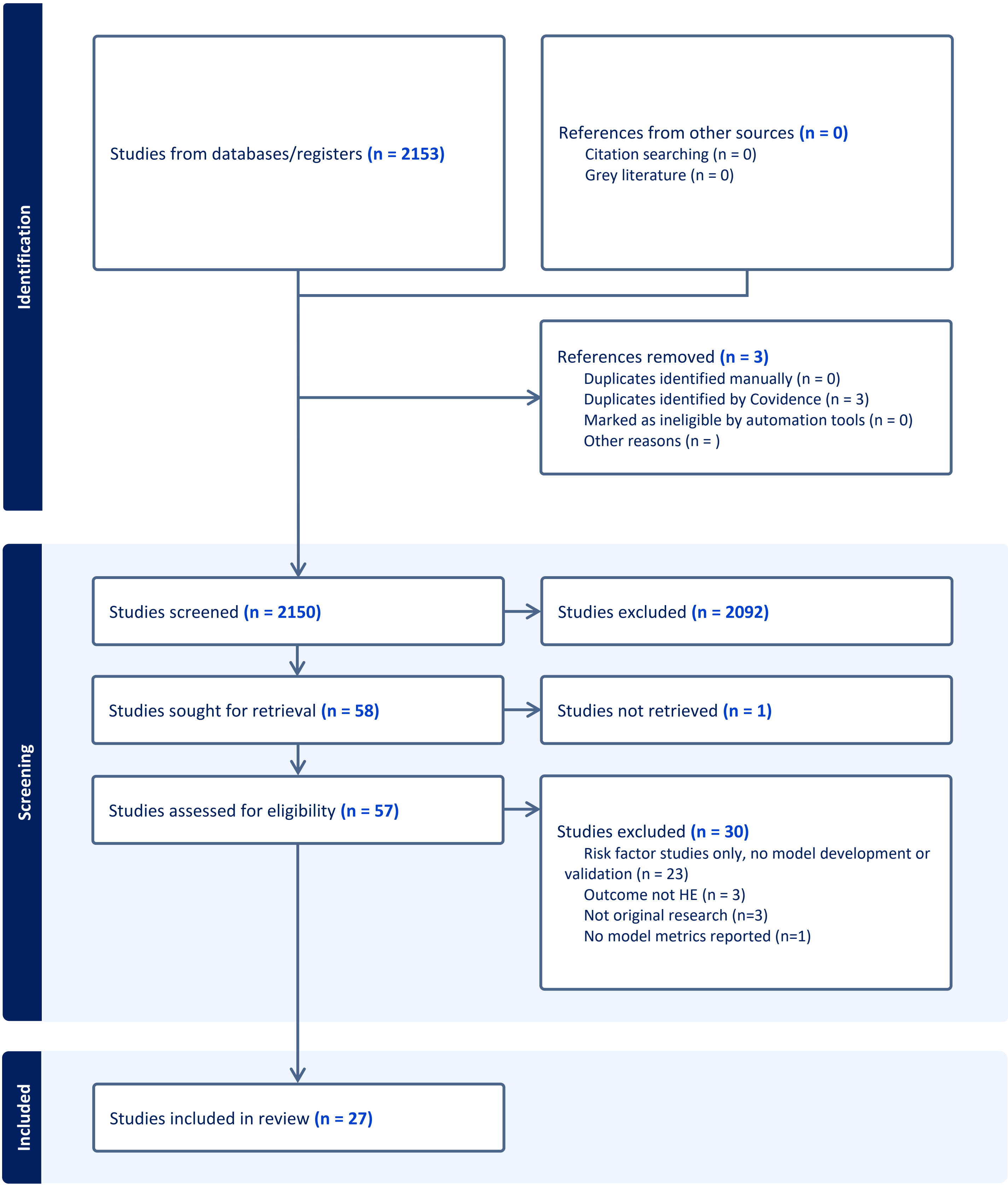

Fig. S2 Discrimination performance of established clinical scores across included studies

Study	Outcome (time window)	Participants/Events (N)	Estimate (range)
**Child-Pugh Score**			
Xu, 2025	severe HE (12-month)	406/78	0.70 (N.R.)
Liao, 2023	OHE (3-month)	207/39	0.66 (0.58–0.76)
Liu, 2021	OHE (NA)	159/37	0.66 (0.56–0.76)
Lin, 2021	HE (12-month)	224/82	0.65 (0.57–0.72)
Cai, 2022	HE (12-month)	241/83	0.64 (N.R.)
Jiang, 2025	OHE (12-month)	310/72	0.64 (0.51–0.76)
Wang, 2021	HE (12-month)	195/45	0.60 (0.51–0.68)
**MELD**			
Jiang, 2025	OHE (12-month)	310/72	0.75 (0.63–0.86)
Miao, 2025	OHE (12-month)	119/23	0.73 (0.51–0.93)
Xu, 2025	severe HE (1-month)	406/78	0.68 (N.R.)
Liao, 2023	OHE (3-month)	207/39	0.66 (0.56–0.76)
Lin, 2021	HE (12-month)	224/82	0.66 (0.58–0.73)
Cai, 2022	HE (12-month)	241/83	0.66 (N.R.)
Chen, 2025	Further decompensation (12 months)	152/52	0.65 (N.R.)
Wang, 2021	HE (12-month)	195/45	0.64 (0.55–0.72)
**MELD-Na**			
Chen, 2025	Further decompensation (12 months)	152/52	0.65 (N.R.)
**MELD 3.0**			
Chen, 2025	Further decompensation (12 months)	152/52	0.63 (N.R.)
**FIPS**			
Xu, 2025	severe HE (1-month)	406/78	0.78 (N.R.)
Cai, 2022	HE (12-month)	241/83	0.74 (0.68–0.80)
Xu, 2025	severe HE (12-month)	406/78	0.70 (N.R.)
**ALBI**			
Lin, 2021	HE (12-month)	224/82	0.74 (0.67–0.81)

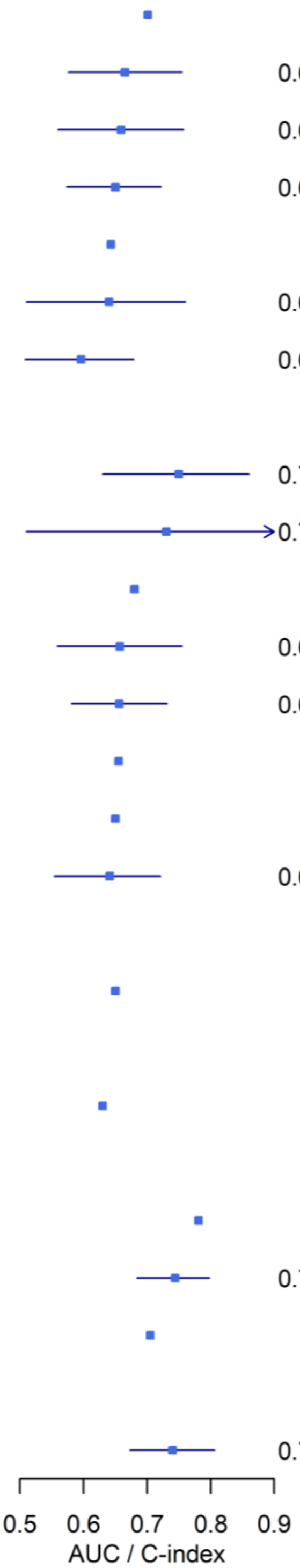

Supplement: Supplementary file 3 [file mmc4.pdf]
