# Supplementary material for: Risk prediction models for hepatic encephalopathy post-transjugular intrahepatic portosystemic shunt: A systematic review and critical appraisal
Source: JHEP Rep. 2026 Aug 22;8(11):102006. doi: 10.1016/j.jhepr.2026.102006 (PMC13694966; doi:10.1016/j.jhepr.2026.102006)
Supplement: Supplementary file 4 [file mmc2.docx]

**JHEP Reports**

**CTAT methods**

Tables for a “Complete, Transparent, Accurate and Timely account” (CTAT) are now mandatory for all revised submissions. The aim is to enhance the reproducibility of methods.

- Only include the parts relevant to your study
- Refer to the CTAT in the main text as ‘Supplementary CTAT Table’
- Do not add subheadings
- Add as many rows as needed to include all information
- Only include one item per row

**If the CTAT form is not relevant to your study, please outline the reasons why:**

Given that ours is a systematic review with no statistical analyses, the CTAT is not applicable. The methodology with regard to the bias assessment is extensively mentioned in the manuscript.
